# Supplementary material for: Exploring Short and Efficient Synthetic Routes Using Titanocene(III)-Catalyzed Reactions: Total Synthesis of Natural Meroterpenes with Trisubstituted Unsaturations
Source: Molecules. 2022 Apr 8;27(8):2400. doi: 10.3390/molecules27082400 (PMC9031794; doi:10.3390/molecules27082400)
Supplement: Supplementary file 1 [file molecules-27-02400-s001.zip › molecules-1665149-supplementary.pdf]

Supporting Information

Exploring short and efficient synthetic routes using titanocene(III)-catalyzed reactions: total synthesis of natural meroterpenes with trisubstituted unsaturations

Jennifer Rosales [1,2], Gustavo Cabrera [2] and José Justicia [1,*]

1. Department of Organic Chemistry, Faculty of Sciences, University of Granada, C. U. Fuentenueva s/n, 18071 Granada, Spain. Fax: +34 958 248437.
2. Centro de Química Orgánica, Escuela de Química, Facultad de Ciencias, Universidad Central de Venezuela, Caracas, Venezuela

E-mail: jjusti@ugr.es

Contents

- Description of new compounds and synthetic processes S2-S9
- ^{1}H and ^{13}C NMR spectra for new compounds S10-S28

Synthesis of allylic alcohols 17 and 20.

The synthesis of compounds **17** and **20** was performed following GP-1.

Compound 17: (hexane: EtOAc 8:2; 92% yield). Colorless oil; ^{1}H NMR (400 MHz, $CDCl_3$) δ 7.13 (dd, *J* = 8.2, 2.4 Hz, 1H), 7.09 (d, *J* = 2.4 Hz, 1H), 6.81 (d, *J* = 8.3 Hz, 1H), 5.59 (t, *J* = 7.2 Hz, 1H), 4.66 (s, 2H), 4.03 (s, 2H), 3.82 (s, 3H), 3.37 (d, *J* = 7.3 Hz, 2H), 1.78 (s, 3H), 1.44 (bs, 1H, OH), 0.94 (s, 9H), 0.09 (s, 6H). ^{13}C NMR (125 MHz, $CDCl_3$) δ 156.5 (C), 135.7 (C), 133.5 (C), 129.1 (C), 127.8 (CH), 125.1 (CH), 124.5 (CH), 110.2 (CH), 69.1 (CH_2), 64.9 (CH_2), 55.6 (CH_3), 28.3 (CH_2), 26.1 (CH_3), 18.3 (C), 13.8 (CH_3), -5.0 (CH_3). HRMS (ESI): m/z $[M+Na]^+$ calcd for $C_{19}H_{32}O_3NaSi$: 359.2018, found: 359.2023.

Compound 20: (hexane: EtOAc 7:3; 99% yield). Colorless oil; Its ^{1}H and ^{13}C NMR spectra matched with previously described [38].

General procedure for oxidation of alcohols with Dess-Martin periodinane (DMP) (GP-4).

To a solution of the corresponding alcohol (1 mmol) in CH_2Cl_2 (20 mL), Dess-Martin periodinane (DMP) (2 mmol) was added, and the mixture was stirred at room temperature for 2-3 h. Then, the solvent was removed, and Et_2O was added. The organic layer was washed with a 1:1 solution of saturated $NaHCO_3$ and 10% $Na_2S_2O_3$, dried (anhyd. Na_2SO_4), and the solvent removed. Products **11** and **12** were purified by flash chromatography on silica gel (mixtures of hexane/EtOAc) and characterized by spectroscopic techniques.

Compound 11: (hexane/EtOAc 9:1; 95% yield). Yellowish oil; ^{1}H NMR (400 MHz, $CDCl_3$) δ 9.41 (s, 1H), 7.18 (dd, *J* = 8.3, 1.8 Hz, 1H), 7.09 (bs, 1H), 6.84 (d, *J* = 8.3 Hz, 1H), 6.62 (t, *J* = 7.4 Hz, 1H), 4.67 (s, 2H), 3.83 (s, 3H), 3.67 (d, *J* = 7.4 Hz, 2H), 1.87 (s, 3H), 0.93 (s, 9H), 0.09 (s, 6H). ^{13}C NMR (125 MHz, $CDCl_3$) δ 195.5 (C), 156.5 (C), 152.8 (CH), 139.4 (C), 133.8 (C), 128.1 (CH), 126.4 (CH), 126.0 (CH), 110.3 (CH), 64.7 (CH_2), 55.6 (CH_3), 30.2 (CH_2), 26.1 (CH_3), 18.5 (C), 9.3 (CH_3), -5.1 (CH_3). HRMS (ESI): m/z $[M+H]^+$ calcd for $C_{19}H_{31}O_3Si$: 335.2042, found: 335.2040.

Compound 12: (hexane/EtOAc 9:1; 96% yield). Colorless oil; Its ^{1}H and ^{13}C NMR spectra matched with previously described [37].

Preparation of hydroxylated polyenes 9 and 10.

The synthesis of compounds **9** and **10** was performed following GP-2.

Compound 9: (hexane/EtOAc 9:1; 51% yield). Colorless oil; ^{1}H NMR (400 MHz, $CDCl_3$) δ 7.13 (dd, *J* = 8.3, 2.1 Hz, 1H), 7.08 (bs, 1H), 6.82 (d, *J* = 8.3 Hz, 1H), 5.58 (t, *J* = 7.3 Hz, 1H), 5.20-5.04 (m, 3H), 4.65 (s, 2H), 4.03 (t, *J* = 6.2 Hz, 1H), 3.81 (s, 3H), 3.36 (d, *J* = 8.8 Hz, 2H), 2.39-2.18 (m, 2H), 2.11-1.93 (m, 8H), 1.74 (s, 3H), 1.68 (s, 3H), 1.64 (s, 3H), 1.60 (s, 3H), 1.59 (s, 3H), 0.94 (s, 9H), 0.08 (s, 6H). ^{13}C NMR (125 MHz, $CDCl_3$) δ 156.4 (C), 138.4 (C), 137.8 (C), 135.2 (C), 133.6 (C), 131.3 (C), 129.1 (C), 127.7 (CH), 125.0 (CH), 124.4 (CH), 124.0 (CH), 120.1 (CH), 110.0 (CH), 77.1 (CH), 64.8 (CH_2), 55.4 (CH_3), 39.9 (CH_2), 39.7 (CH_2), 34.2 (CH_2), 28.1 (CH_2), 26.8 (CH_2), 26.6 (CH_2), 26.0 (CH_3), 25.7 (CH_3), 18.4 (C), 17.7 (CH_3), 16.3 (CH_3), 16.0 (CH_3), 11.8 (CH_3), -5.1 (CH_3). HRMS (ESI): m/z $[M+Na]^+$ calcd for $C_{34}H_{56}O_3NaSi$: 563.3896, found: 563.3910.

Compound 10: (hexane/EtOAc 9:1; 60% yield). Colorless oil; ^{1}H NMR (400 MHz, $CDCl_3$) δ 6.78-6.72 (m, 2H), 6.71-6.66 (m, 1H), 5.57 (t, *J* = 7.3 Hz, 1H), 5.19-5.05 (m, 3H), 4.04 (t, *J* = 7.6 Hz, 1H), 3.78 (s, 3H), 3.75 (s, 3H), 3.35 (d, *J* = 7.3 Hz, 2H), 2.41-2.22 (m, 2H), 2.14-2.02 (m, 6H), 2.01-1.94 (m, 2H), 1.74 (s, 3H), 1.69 (s, 3H), 1.64 (s, 3H), 1.61 (s, 6H). ^{13}C NMR (125 MHz, $CDCl_3$) δ 153.6 (C), 151.7 (C), 138.4 (C), 138.0 (C), 135.2 (C), 131.3 (C), 130.8 (C), 124.5 (CH), 124.2 (CH), 124.1 (CH), 120.1 (CH), 116.2 (CH), 111.2 (CH), 110.7 (CH), 77.2 (CH), 56.0 (CH_3), 55.7 (CH_3), 39.9 (CH_2), 39.8 (CH_2), 34.2 (CH_2), 28.2 (CH_2), 26.8 (CH_2), 26.6 (CH_2), 25.8 (CH_3), 17.7 (CH_3), 16.4 (CH_3), 16.1 (CH_3), 11.8 (CH_3). HRMS (ESI): m/z $[M+Na]^+$ calcd for $C_{28}H_{42}O_3Na$: 449.3032, found: 449.3039.

General procedure for acetylation of alcohols (GP-5).

To a solution of corresponding alcohol (1 mmol) in CH_2Cl_2 (25 mL), Ac_2O (2 mmol) and DMAP (2 mmol) were added, and the mixture was stirred for 2-6 h at room temperature. Then, the solvent was removed. The residue was purified by flash chromatography on silica gel (mixtures hexane/EtOAc) to yield the corresponding acetate.

Compound 18: (hexane/EtOAc 95:5; 99% yield). Colorless oil; ^{1}H NMR (400 MHz, $CDCl_3$) δ 7.14 (dd, *J* = 8.3, 2.1 Hz, 1H), 7.04 (d, *J* = 2.1 Hz, 1H), 6.79 (d, *J* = 8.3 Hz, 1H), 5.61 (t, *J* = 7.3 Hz, 1H), 5.17 (t, *J* = 6.9 Hz, 1H), 5.12-4.91 (m, 2H), 4.65 (s, 2H), 3.81 (s, 3H), 3.35 (d, *J* = 7.3 Hz, 2H), 2.48-2.36 (m, 2H), 2.33-2.21 (m, 2H), 2.09-2.02 (m, 4H), 2.02 (s, 3H), 2.00-1.91 (m, 2H), 1.73 (s, 3H), 1.68 (s, 3H), 1.60 (s, 6H), 1.58 (s, 3H), 0.93 (s, 9H), 0.08 (s, 6H). HRMS (ESI): m/z $[M+Na]^+$ calcd for $C_{36}H_{58}O_4NaSi$: 605.4002, found: 605.4011.

Compound 21: (hexane/EtOAc 95:5; 99% yield). Colorless oil; ^{1}H NMR (400 MHz, $CDCl_3$) δ 6.78-6.67 (m, 3H), 5.63 (t, *J* = 7.4 Hz, 1H), 5.19 (t, *J* = 7.0 Hz, 1H), 5.11 (t, *J* = 7.0 Hz, 1H), 5.06 (t, *J* = 7.0, 1H), 3.77 (s, 3H), 3.75 (s, 3H), 3.35 (d, *J* = 7.4 Hz, 2H), 2.49-2.38 (m, 2H), 2.37-2.27 (m, 2H), 2.12-2.05 (m, 3H), 2.04 (s, 3H), 2.01-1.96 (m, 3H), 1.73 (s, 3H), 1.69 (s, 3H), 1.63 (s, 3H), 1.62 (s, 3H), 1.60 (s, 3H). ^{13}C NMR (125 MHz, $CDCl_3$) δ 170.2 (C), 153.6 (C), 151.6 (C), 137.8 (C), 135.0 (C), 134.1 (C), 131.2 (C), 130.3 (C), 126.2 (CH), 124.4 (CH), 124.2 (CH), 119.1 (CH), 115.8 (CH), 111.1 (CH), 110.9 (CH), 79.0 (CH), 55.9 (CH_3), 55.6 (CH_3), 39.8 (CH_2), 31.6 (CH_2), 28.0 (CH_2), 26.8 (CH_2), 26.6 (CH_2), 25.7 (CH_3), 21.3 (CH_3), 17.7 (CH_3), 16.3 (CH_3), 16.0 (CH_3), 12.1 (CH_3). HRMS (ESI): m/z $[M+Na]^+$ calcd for $C_{30}H_{44}O_4Na$: 491.3137, found: 491.3141.

General procedure for the epoxidation of compounds 18 and 21 (GP-6).

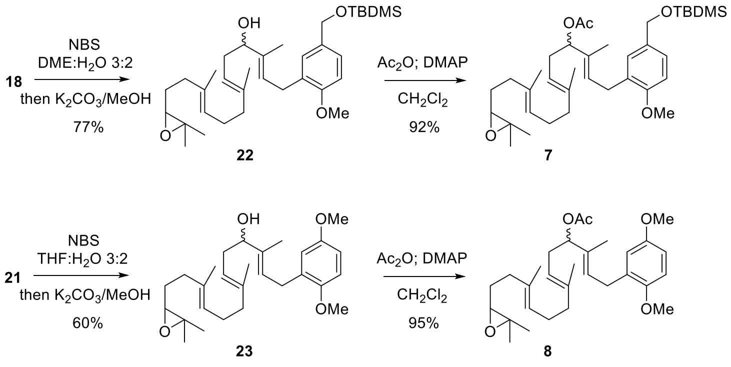

To a solution of corresponding polyene (1 mmol) in a 3:2 mixture of DME:H_2O (50 mL, for compound **18**) or a 3:2 mixture of THF:H_2O (50 mL, for compound **21**), NBS (1 mmol) was added. The mixture was stirred at 0 ºC for 15-30 minutes (followed by TLC). Then, Et_2O was added, and the mixture was washed with water, dried (anhyd. Na_2SO_4), and the solvent was removed. The residue was dissolved in methanol (20 mL), K_2CO_3 (5 mmol) was added, and the mixture was stirred overnight. Then, the solvent was removed, Et_2O was added, and the mixture was washed with brine, dried (anhyd. Na_2SO_4), and the solvent removed. The residue was submitted to flash chromatography on silica gel (mixtures of hexane/EtOAc) to yield the corresponding epoxides **22** (77%) and **23** (60%).

Compound 22: (hexane/EtOAc 9:1). Yellowish oil; ^{1}H NMR (400 MHz, CDCl3) δ 7.13 (dd, *J* = 8.3, 2.0 Hz, 1H), 7.07 (s, 1H), 6.80 (d, *J* = 8.3 Hz, 1H), 5.57 (t, *J* = 7.3 Hz, 1H), 5.20-5.08 (m, 2H), 4.65 (s, 2H), 4.02 (dd, *J* = 7.4, 5.7 Hz, 1H), 3.81 (s, 3H), 3.36 (d, *J* = 7.3 Hz, 2H), 2.69 (t, *J*

= 6.2 Hz, 1H), 2.36-2.19 (m, 2H), 2.19-1.99 (m, 6H), 1.74 (s, 3H), 1.63 (s, 3H), 1,61 (s, 3H), 1.29 (s, 3H), 1.25 (s, 3H), 0.93 (s, 9H), 0.08 (s, 6H). ^{13}C NMR (125 MHz, $CDCl_3$) δ 156.5 (C), 138.1 (C), 138.0 (C), 134.3 (C), 133.3 (C), 129.2 (C), 127.7 (CH), 125.0 (CH), 124.8 (CH), 124.3 (CH), 120.4 (CH), 110.1 (CH), 77.2 (CH), 64.9 (CH_2), 64.2 (CH), 58.4 (C), 55.5 (CH_3), 39.8 (CH_2), 36.4 (CH_2), 34.2 (CH_2), 28.2 (CH_2), 27.5 (CH_2), 26.6 (CH_2), 26.0 (CH_3), 25.0 (CH_3), 18.8 (CH_3), 18.5 (C), 16.4 (CH_3), 16.1 (CH_3), 11.8 (CH_3), -5.1 (CH_3). HRMS (ESI): m/z $[M+Na]^+$ calcd for $C_{34}H_{56}O_4NaSi$: 579.3846, found: 579.3856.

Compound 23: (hexane/EtOAc 7:3). Yellowish oil. This compound was used immediately in the next step without further purification.

From alcohols **22** and **23**, compounds **7** and **8** were prepared following GP-5.

Compound 7: (hexane/EtOAc 9:1, 92%). Yellowish oil; ^{1}H NMR (400 MHz, $CDCl_3$) δ 7.13 (dd, *J* = 8.3, 2.0 Hz, 1H), 7.03 (bs, 1H), 6.79 (d, *J* = 8.3 Hz, 1H), 5.60 (t, *J* = 7.2 Hz, 1H), 5.21-5.10 (m, 2H), 5.02 (t, *J* = 7.2 Hz, 1H), 4.64 (s, 2H), 3.80 (s, 3H), 3.34 (d, *J* = 7.2 Hz, 2H), 2.69 (t, *J* = 6.2 Hz, 1H), 2.50-2.20 (m, 3H), 2.18-1.92 (m, 5H), 2.02 (s, 3H), 1.72 (s, 3H), 1.69-1.61 (m, 2H), 1.60 (s, 6H), 1.29 (s, 3H), 1.25 (s, 3H), 0.93 (s, 9H), 0.08 (s, 6H). ^{13}C NMR (125 MHz, $CDCl_3$) δ 170.4 (C), 156.5 (C), 137.7 (C), 134.2 (C), 133.8 (C), 133.4 (C), 128.9 (C), 127.8 (CH), 126.5 (CH), 125.2 (CH), 124.8 (CH), 119.4 (CH), 110.1 (CH), 79.1 (CH), 65.0 (CH_2), 64.3 (CH), 58.4 (C), 55.5 (CH_3), 39.8 (CH_2), 36.4 (CH_2), 31.7 (CH_2), 28.2 (CH_2), 27.6 (CH_2), 26.7 (CH_2), 26.0 (CH_3), 25.0 (CH_3), 21.4 (CH_3), 18.8 (CH_3), 18.6 (C), 16.3 (CH_3), 16.1 (CH_3), 12.3 (CH_3), -5.0 (CH_3). HRMS (ESI): m/z $[M+Na]^+$ calcd for $C_{36}H_{58}O_5NaSi$: 621.3951, found: 621.3960.

Compound 8: (hexane/EtOAc 9:1, 95%). Yellowish oil; ^{1}H NMR (400 MHz, $CDCl_3$) δ 6.79-6.72 (m, 1H), 6.71-6.64 (m, 2H), 5.60 (t, *J* = 7.3 Hz, 1H), 5.19-5.10 (m, 2H), 5.03 (t, *J* = 6.8 Hz, 1H), 3.77 (s, 3H), 3.74 (s, 3H), 3.33 (d, *J* = 7.4 Hz, 2H), 2.69 (t, *J* = 6.2 Hz, 1H), 2.46-2.35 (m, 2H), 2.34-1.24 (m, 2H), 2.19-2.04 (m, 2H), 2.03 (s, 3H), 2.01-1.93 (m, 2H) 1.70 (s, 3H), 1.70-1.62 (m, 2H), 1.60 (s, 6H), 1.29 (s, 3H), 1.25 (s, 3H). ^{13}C NMR (125 MHz, $CDCl_3$) δ 170.0 (C), 153.5 (C), 151.5 (C), 137.5 (C), 134.0 (C), 133.9 (C), 130.1 (C), 126.0 (C), 124.7 (CH), 119.1 (CH), 115.6 (CH), 110.9 (CH), 110.8 (CH), 78.9 (CH), 63.9 (CH), 58.0 (C), 55.7 (CH_3), 55.4 (CH_3), 39.5 (CH_2), 36.2 (CH_2), 31.5 (CH_2), 27.9 (CH_2), 27.4 (CH_2), 26.5 (CH_2), 24.8 (CH_3), 21.1 (CH_3), 18.6 (CH_3), 16.1 (CH_3), 15.8 (CH_3), 12.0 (CH_3). HRMS (ESI): m/z $[M+Na]^+$ calcd $C_{30}H_{44}O_5Na$: 507.3086, found: 507.3082.

Synthesis of polycyclic compounds 5 and 6 using Cp_2TiCl-catalyzed cyclization reactions. The synthesis of compounds **5** and **6** was performed following GP-3.

Compound 5: (hexane/EtOAc 9:1; 43% yield). Colorless oil; ^{1}H NMR (400 MHz, $CDCl_3$) δ 7.22 (bs, 1H), 7.06 (dd, *J* = 8.3, 1.8 Hz, 1H), 6.78 (d, *J* = 8.3 Hz, 1H), 5.31 (bs, 1H), 4.67 (s, 2H), 3.81 (s, 3H), 3.22 (dd, *J* = 11.0, 5.2 Hz, 1H), 2.75 (dd, *J* = 15.3, 9.4 Hz, 1H), 2.56 (d, *J* = 15.3 Hz, 1H), 2.42-2.35 (m, 1H), 2.07-1.97 (m, 2H), 1.95-1.86 (m, 3H), 1.76-1.52 (m, 7H), 1.42 (s, 3H), 1.00 (s, 3H), 0.94 (s, 9H), 0.92 (s, 3H), 0.88 (s, 3H), 0.80 (s, 3H), 0.08 (s, 6H). ^{13}C NMR (125 MHz, $CDCl_3$) δ 156.3 (C), 136.0 (C), 133.2 (C), 132.0 (C), 127.6 (CH), 124.1 (CH), 121.5 (CH), 110.2 (CH), 79.2 (CH), 64.9 (CH_2), 55.5 (CH_3), 55.42 (CH), 55.40 (CH), 55.0 (CH), 41.3 (CH_2), 38.9 (C), 38.5 (CH_2), 37.2 (C), 37.0 (C), 28.3 (CH_3), 27.4 (CH_2), 26.1 (CH_3), 26.0 (CH_2), 23.0 (CH_2), 22.1 (CH_3), 18.8 (CH_2), 18.5 (C), 15.8 (CH_3), 15.7 (CH_3), 14.8 (CH_3), -5.0 (CH_3). HRMS (ESI): m/z $[M+Na]^+$ calcd for $C_{34}H_{56}O_3NaSi$: 563.3896, found: 563.3914.

Compound 6: (hexane/EtOAc 9:1; 35% yield). Colorless oil; ^{1}H NMR (400 MHz, $CDCl_3$) δ 6.83 (d, *J* = 2.9 Hz, 1H), 6.73 (d, *J* = 8.8 Hz, 1H), 6.66 (dd, *J* = 8.8, 2.9 Hz, 1H), 5.33 (bs, 1H), 3.78 (s, 3H), 3.77 (s, 3H), 3.22 (dd, *J* = 11.1, 5.1 Hz, 1H), 2.73 (dd, *J* = 15.4, 9.5 Hz, 1H), 2.54 (d, *J* = 15.4 Hz, 1H), 2.39-2.32 (m, 1H), 2.01 (dt, *J* = 13.9, 3.0 Hz, 1H), 1.95-1.88 (m, 2H), 1.73-1.52 (m, 6H), 1.43 (s, 3H), 1.34-1.15 (m, 2H), 1.00 (s, 3H), 0.91 (s, 3H) , 0.87 (s, 3H) , 0.80 (s, 3H). ^{13}C NMR (125 MHz, $CDCl_3$) δ 153.6 (C), 151.7 (C), 135.8 (C), 133.8 (C), 121.7 (CH), 116.4 (CH), 111.3 (CH), 110.0 (CH), 79.2 (CH), 56.0 (CH), 55.8 (CH), 55.4 (CH_3), 55.3 (CH), 54.9 (CH_3), 41.3 (CH_2), 38.9 (CH_2), 38.5 (C), 37.2 (C), 37.0 (C), 28.3 (CH_3), 27.4 (CH_2), 26.2 (CH_2), 23.0 (CH_2), 22.2 (CH_3), 18.8 (CH_2), 15.8 (CH_3), 15.7 (CH_3), 14.8 (CH_3). HRMS (ESI): m/z $[M+Na]^+$ calcd for $C_{28}H_{42}O_3Na$: 449.3032, found: 449.3016.

General procedure for Barton-McCombie deoxygenation: Step A: To a solution of corresponding alcohol (1 mmol) in 1,2-dichloroethane (DCE) (20 mL), DMAP (3 mmol), Et_3N (1.5 mmol) and pentafluorophenyl chlorothionoformate (2 mmol) were added, and the mixture was stirred for 2-4 h at room temperature under an Ar atmosphere. Then, the solvent was removed. The residue was purified by flash chromatography on silica gel (mixtures of hexane/EtOAc).

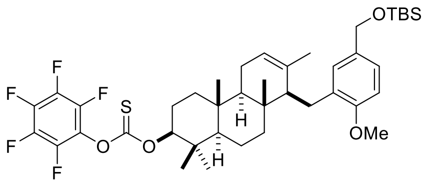

24

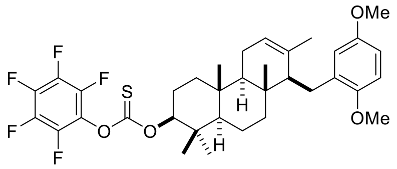

25

Compound 24: (hexane/EtOAc 98:2; 57% yield). Yellow oil; ^{1}H NMR (400 MHz, $CDCl_3$) δ 7.20 (bs, 1H), 7.08 (d, *J* = 8.3 Hz, 1H), 6.78 (d, *J* = 8.3 Hz, 1H), 5.34 (bs, 1H), 4.96 (dd, *J* = 11.8, 4.5 Hz, 1H), 4.68 (s, 2H), 3.82 (s, 3H), 2.76 (dd, *J* = 15.2, 9.2 Hz, 1H), 2.57 (d, *J* = 15.2 Hz, 1H), 2.44-2.37 (m, 1H), 2.10-1.53 (m, 11H), 1.44 (s, 3H), 1.01 (s, 3H), 0.99 (s, 3H), 0.97 (s, 3H), 0.94 (s, 9H), 0.90 (s, 3H), 0.09 (s, 6H). ^{13}C NMR (125 MHz, $CDCl_3$) δ 191.8 (C), 156.3 (C), 136.0 (C), 133.3 (C), 131.9 (C), 127.7 (CH), 124.2 (CH), 121.3 (CH), 110.3 (CH), 95.5 (CH), 65.0 (CH_2), 55.5 (CH_3), 55.4 (CH), 55.2 (CH), 54.8 (CH), 41.1 (CH_2), 38.7 (C), 38.0 (CH_2), 37.2 (C), 37.1 (C), 29.8 (CH_2), 28.0 (CH_3), 26.1 (CH_2), 26.0 (CH_3), 23.0 (CH_2), 22.3 (CH_2), 22.2 (CH_3), 18.6 (CH_2), 18.5 (C), 17.1 (CH_3), 15.9 (CH_3), 14.7 (CH_3), -5.0 (CH_3) (some C signals were not observed). HRMS (ESI): m/z $[M+Na]^+$ calcd for $C_{41}H_{55}O_4NaSSiF_5$: 789.3408, found: 789.3416.

Compound 25: (hexane/EtOAc 98:2; 53% yield). Yellow oil; ^{1}H NMR (400 MHz, $CDCl_3$) δ 6.82 (d, *J* = 3.0 Hz, 1H) 6.75 (d, *J* = 8.8 Hz, 1H), 6.66 (dd, *J* = 8.8, 3.0 Hz, 1H), 5.33 (bs, 1H), 4.95 (dd, *J* = 11.8, 4.6 Hz, 1H), 3.78 (s, 3H), 3.77 (s, 3H), 2.74 (dd, *J* = 15.3, 9.4 Hz, 1H), 2.55 (d, *J* = 15.3 Hz, 1H), 2.40-2.35 (m, 1H), 2.01 (dt, *J* = 13.9, 3.0 Hz, 1H), 2.00-1.90 (m, 2H), 1.85-1.75 (m, 2H), 1.63-1.54 (m, 2H), 1.44 (s, 3H), 1.37-1.23 (m, 4H), 1.00 (s, 3H), 0.99 (s, 3H), 0.97 (s, 3H), 0.89 (s, 3H). ^{13}C NMR (125 MHz, $CDCl_3$) δ 191.8 (C), 153.5 (C), 151.6 (C), 135.8 (C), 133.5 (C), 121.5 (CH), 116.3 (CH), 111.3 (CH), 110.1 (CH), 95.4 (CH), 56.0 (CH), 55.8 (CH), 55.4 (CH_3), 55.1 (CH), 54.7 (CH_3), 41.0 (CH_2), 38.7 (C), 38.0 (CH_2), 37.1 (C), 37.0 (C), 31.9 (C), 28.0 (CH_3), 26.3 (CH_2), 23.0 (CH_2), 22.3 (CH_2), 22.2 (CH_3), 18.6 (CH_2), 17.1 (CH_3), 15.9 (CH_3), 14.8 (CH_3) (some carbon signals were no observed). HRMS (ESI): m/z $[M+Na]^+$ calcd for $C_{35}H_{41}O_5F_5NaS$: 691.2493, found: 691.2465.

Step B: To a solution of corresponding compound (1 mmol) in benzene (40 mL), AIBN (0.2 mmol) and $HSnBu_3$ (3 mmol) were added under an Ar atmosphere. The mixture was stirred under reflux (85 ºC) for 4h. Then, the solvent was removed, and the residue was purified by flash chromatography on silica gel (mixtures of hexane/AcOEt).

Compound 19: (hexane/EtOAc 99:1; 91% yield). Colorless oil; ^{1}H NMR (400 MHz, $CDCl_3$) δ 7.23 (bs, 1H), 7.06 (d, *J* = 8.3 Hz, 1H), 6.78 (d, *J* = 8.3 Hz, 1H), 5.33 (bs, 1H), 4.68 (s, 2H), 3.81 (s, 3H), 2.74 (dd, *J* = 15.3, 9.5 Hz, 1H), 2.55 (d, *J* = 15.3 Hz, 1H), 2.41-2.36 (m, 1H), 2.01 (dt, *J* = 12.8, 3.1 Hz, 1H), 1.94-1.87 (m, 2H), 1.68-1.51 (m, 4H), 1.42 (s, 3H), 1.39-1.29 (m, 4H), 1.25-1.08 (m, 3H), 0.94 (s, 9H), 0.91 (s, 3H), 0.87 (s, 6H), 0.83 (s, 3H), 0.08 (s, 6H). ^{13}C NMR (125 MHz, $CDCl_3$) δ 156.2 (C), 136.0 (C), 133.2 (C), 132.2 (C), 127.6 (CH), 124.0 (CH), 121.8 (CH), 110.3 (CH), 64.9 (CH_2), 56.4 (CH), 55.6 (CH_3), 55.5 (CH), 55.2 (CH), 42.1 (CH_2), 41.3 (CH_2), 40.1 (CH_2), 37.5 (C), 37.1 (C), 33.6 (CH_3), 33.3 (C), 26.1 (CH_3), 25.8 (CH_2), 22.9

(CH_2), 22.1 (CH_3), 21.9 (CH_3), 19.1 (CH_2), 18.7 (CH_2), 18.6 (C), 15.8 (CH_3), 14.8 (CH_3), -5.0 (CH_3). HRMS (ESI): m/z $[M+Na]^+$ calcd for $C_{34}H_{56}O_2NaSi$: 547.3947, found: 547.3954.

Compound 4: (hexane/EtOAc 99:1; 99% yield). Colorless oil; ^{1}H NMR (400 MHz, $CDCl_3$) δ 6.85 (d, *J* = 2.9 Hz, 1H), 6.75 (d, *J* = 8.8 Hz, 1H), 6.66 (dd, *J* = 8.8, 2.9 Hz, 1H), 5.34 (bs, 1H), 3.78 (s, 3H), 3.77 (s, 3H), 2.73 (dd, *J* = 15.4, 9.5 Hz, 1H), 2.55 (dd, *J* = 15.4 Hz, 1H), 2.40-2.33 (m, 1H), 2.00 (dt, *J* = 12.8, 3.0 Hz, 2H), 1.95-1.85 (m, 2H), 1.69-1.46 (m, 4H), 1.43 (s, 3H), 1.39-1.08 (m, 4H), 0.91 (s, 3H), 0.87 (s, 6H) , 0.83 (s, 3H). ^{13}C NMR (125 MHz, $CDCl_3$) δ 153.6 (C), 151.7 (C), 135.8 (C), 134.0 (C), 122.0 (CH), 116.4 (CH), 111.3 (CH), 110.0 (CH), 56.4 (CH), 56.0 (CH), 55.7 (CH_3), 55.4 (CH), 54.9 (CH_3), 42.1 (CH_2), 41.3 (CH_2), 40.1 (CH_2), 37.5 (C), 37.1 (C), 33.6 (CH), 33.3 (C), 31.9 (CH_3), 26.0 (CH_2), 22.9 (CH_2), 22.2 (CH_3), 21.9 (CH_3), 19.1 (CH_2), 18.7 (CH_2), 15.8 (CH_3), 14.9 (CH_3). HRMS (ESI): m/z $[M+Na]^+$ calcd $C_{28}H_{42}O_2Na$: 433.3083, found: 433.3062.

Synthesis of 3.

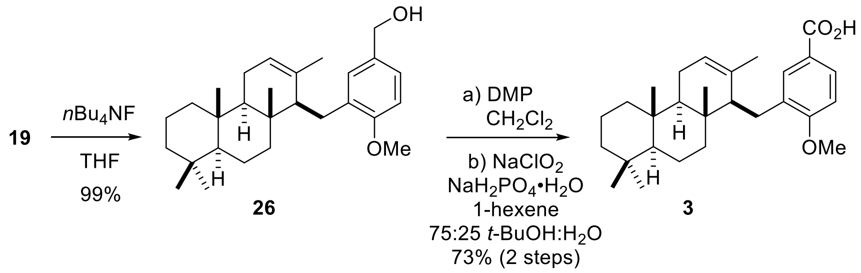

Preparation of intermediate 26: To a solution of compound **19** (39 mg, 0.08 mmol) in THF (4 mL) under an Ar atmosphere, *n*Bu_4NF (1M in THF, 0.19 mL, 0.19 mmol) was added, and the mixture was stirred at room temperature for 4 h. Then, AcOEt was added and the mixture was washed with saturated solution of NH_4Cl, and brine, dried (anhyd. Na_2SO_4), and the solvent was removed. The residue was purified by flash chromatography on silica gel (hexane/EtOAc 85:15) to yield alcohol **26** (30 mg, 99%).

Compound 26: Colorless oil; ^{1}H NMR (400 MHz, CDCl3) δ 7.26 (bs, 1H), 7.13 (dd, *J* = 8.3, 2.0 Hz, 1H), 6.80 (d, *J* = 8.3 Hz, 1H), 5.34 (bs, 1H), 4.61 (s, 2H), 3.82 (s, 3H), 2.76 (dd, *J* = 15.5, 9.8 Hz, 1H), 2.56 (d, *J* = 15.5 Hz, 1H), 2.43-2.37 (m, 1H), 2.00 (dt, *J* = 12.8, 3.1 Hz, 1H), 1.94-1.87 (m, 2H), 1.67-1.51 (m, 5H), 1.39 (s, 3H), 1.37-1.09 (m, 6H), 0.91 (s, 3H), 0.88 (s, 6H), 0.83 (s, 3H). ^{13}C NMR (125 MHz, $CDCl_3$) δ 156.9 (C), 135.7 (C), 132.8 (C), 132.7 (C), 128.7 (CH), 125.5 (CH), 122.1 (CH), 110.4 (CH), 65.6 (CH_2), 56.3 (CH), 55.6 (CH_3), 55.4 (CH), 54.8 (CH), 42.1 (CH_2), 41.3 (CH_2), 40.1 (CH_2), 37.5 (C), 37.1 (C), 33.6 (CH_3), 33.3 (C),

25.7 (CH_2), 22.9 (CH_2), 22.2 (CH_3), 21.9 (CH_3), 19.1 (CH_2), 18.7 (CH_2), 15.8 (CH_3), 14.9 (CH_3). HRMS (ESI): m/z $[M+Na]^+$ calcd for $C_{28}H_{42}O_2Na$: 433.3083, found: 433.3079.

Synthesis of 3. Following GP-4, aldehyde **27** was obtained, and used immediately in the next step without purification.

To a solution of corresponding aldehyde (0.08 mmol) and 1-hexene (9.8 mg, 0.12 mmol) in a 7.5:2.5 mixture of *t*-BuOH:H_2O (5.3 mL), a solution of $NaClO_2$ (71 mg, 0.62 mmol), and $NaH_2PO_4{\cdot}H_2O$ (65 mg, 0.47 mmol) in water (1.4 mL) was slowly added. The mixture was stirred for 3 h at room temperature. Then, AcOEt was added and the mixture was washed with brine, dried (anhyd. Na_2SO_4), and the solvent was removed. The residue was purified by flash chromatography on silica gel (hexane/EtOAc 6:4) to yield acid **3** (24 mg, 73%, 2 steps).

Compound 3: Colorless oil; ^{1}H NMR (400 MHz, $CDCl_3$) δ 8.01 (bs, 1H), 7.93 (dd, *J* = 8.5, 2.1 Hz, 1H), 6.87 (d, *J* = 8.5 Hz, 1H), 5.35 (bs, 1H), 3.90 (s, 3H), 2.76 (dd, *J* = 15.7, 9.7 Hz, 1H), 2.59 (d, *J* = 15.7 Hz, 1H), 2.48-2.41 (m, 1H), 1.98 (dt, *J* = 12.8, 3.1 Hz, 1H), 1.95-1.84 (m, 2H), 1.69-1.52 (m, 5H), 1.38 (s, 3H), 1.34-1.10 (m, 6H), 0.92 (s, 3H), 0.88 (s, 6H), 0.83 (s, 3H). ^{13}C NMR (125 MHz, $CDCl_3$) δ 171.0 (C), 161.8 (C), 135.3 (C), 132.7 (C), 131.6 (CH), 129.7 (CH), 122.4 (CH), 109.9 (CH), 56.2 (CH), 55.7 (CH_3), 55.3 (CH), 54.4 (CH), 42.1 (CH_2), 41.2 (CH_2), 40.1 (CH_2), 37.5 (C), 37.2 (C), 33.6 (CH_3), 33.3 (C), 25.8 (CH_2), 22.9 (CH_2), 22.3 (CH_3), 21.9 (CH_3), 19.1 (CH_2), 18.7 (CH_2), 15.8 (CH_3), 14.9 (CH_3). HRMS (ESI): m/z $[M-H]^+$ calcd for $C_{28}H_{39}O_3$: 423.2899, found: 423.2891.

^{1}H and ^{13}C NMR FOR NEW COMPOUNDS

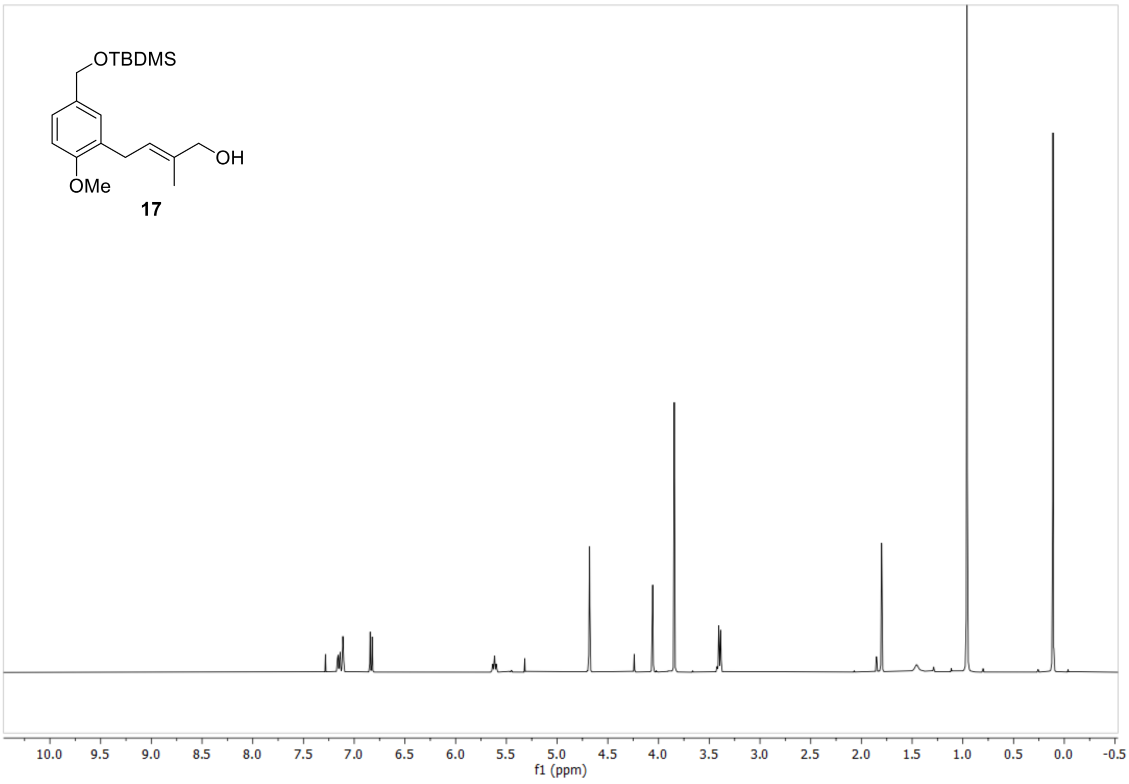

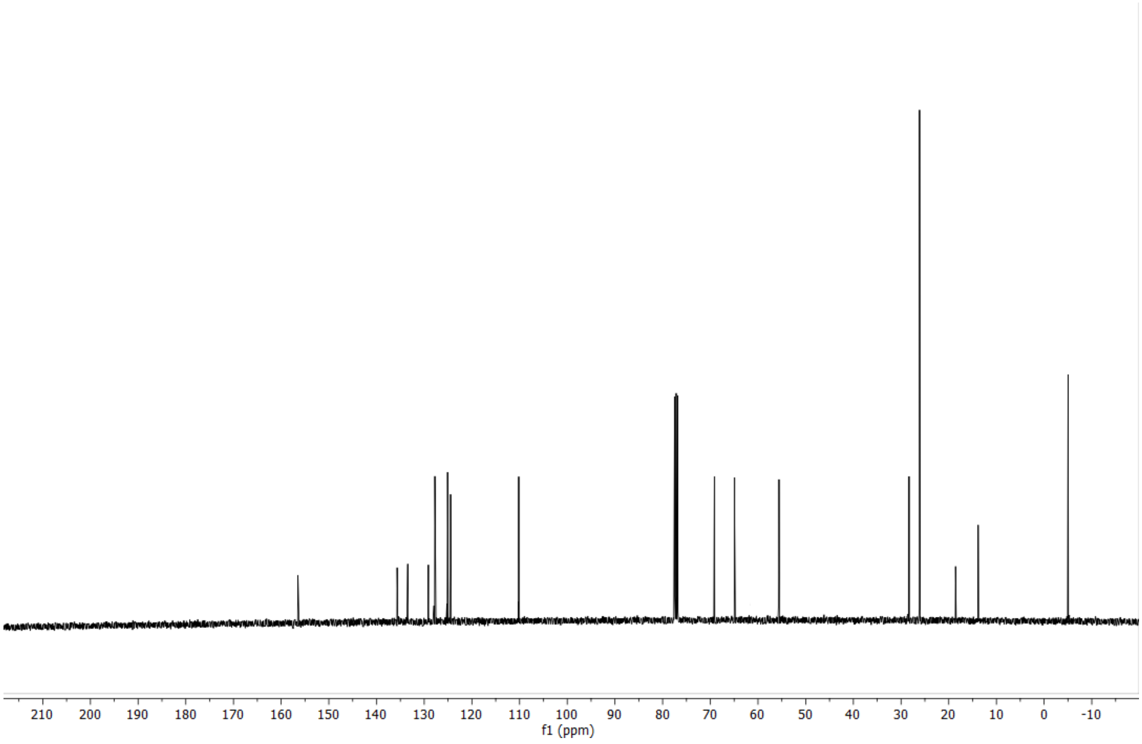

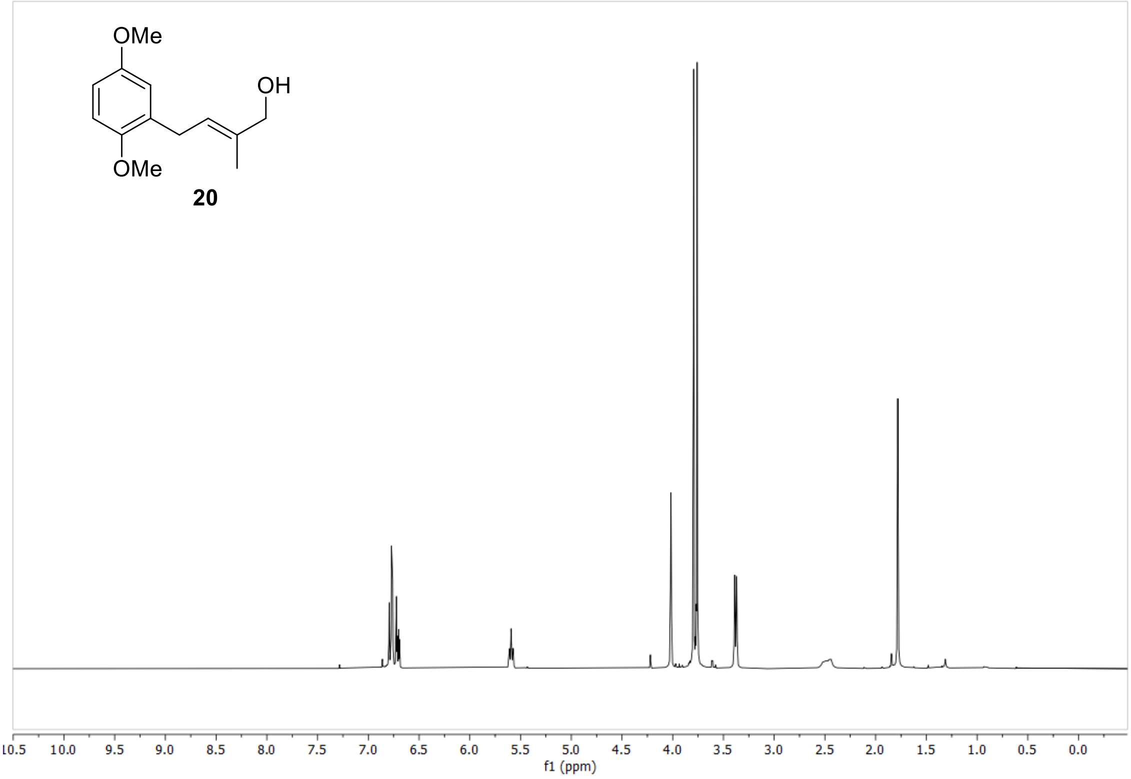
OMe
OH
OMe
20
10.5
10.0
9.5
9.0
8.5
8.0
7.5
7.0
6.5
6.0
5.5
5.0
4.5
4.0
3.5
3.0
2.5
2.0
1.5
1.0
0.5
0.0
f1 (ppm)

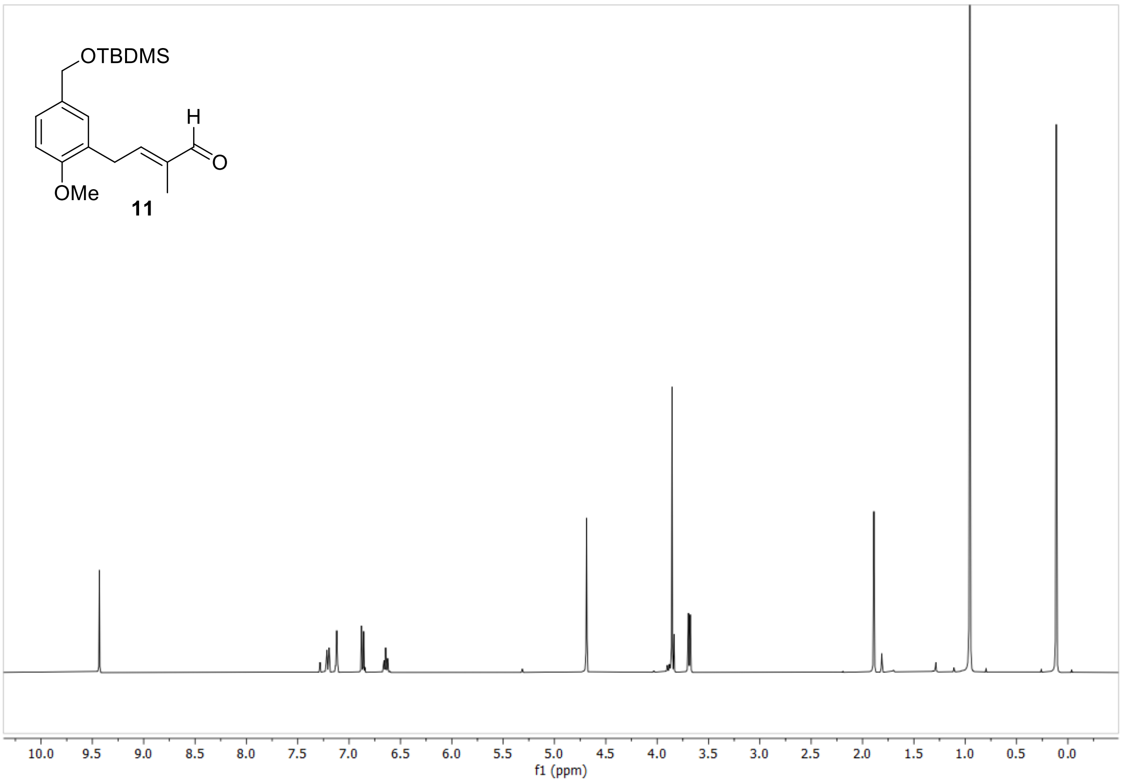
OTBDMS
H
O
OMe
11
10.0 9.5 9.0 8.5 8.0 7.5 7.0 6.5 6.0 5.5 5.0 4.5 4.0 3.5 3.0 2.5 2.0 1.5 1.0 0.5 0.0
f1 (ppm)

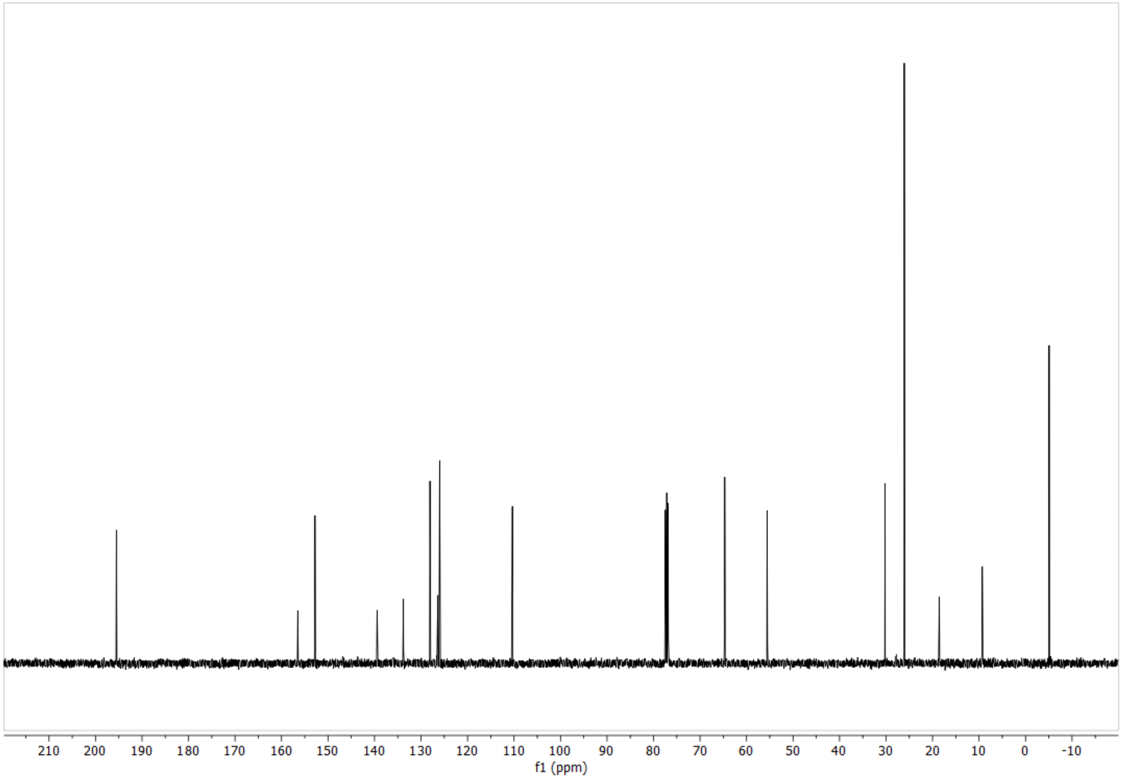
210 200 190 180 170 160 150 140 130 120 110 100 90 80 70 60 50 40 30 20 10 0 -10
f1 (ppm)

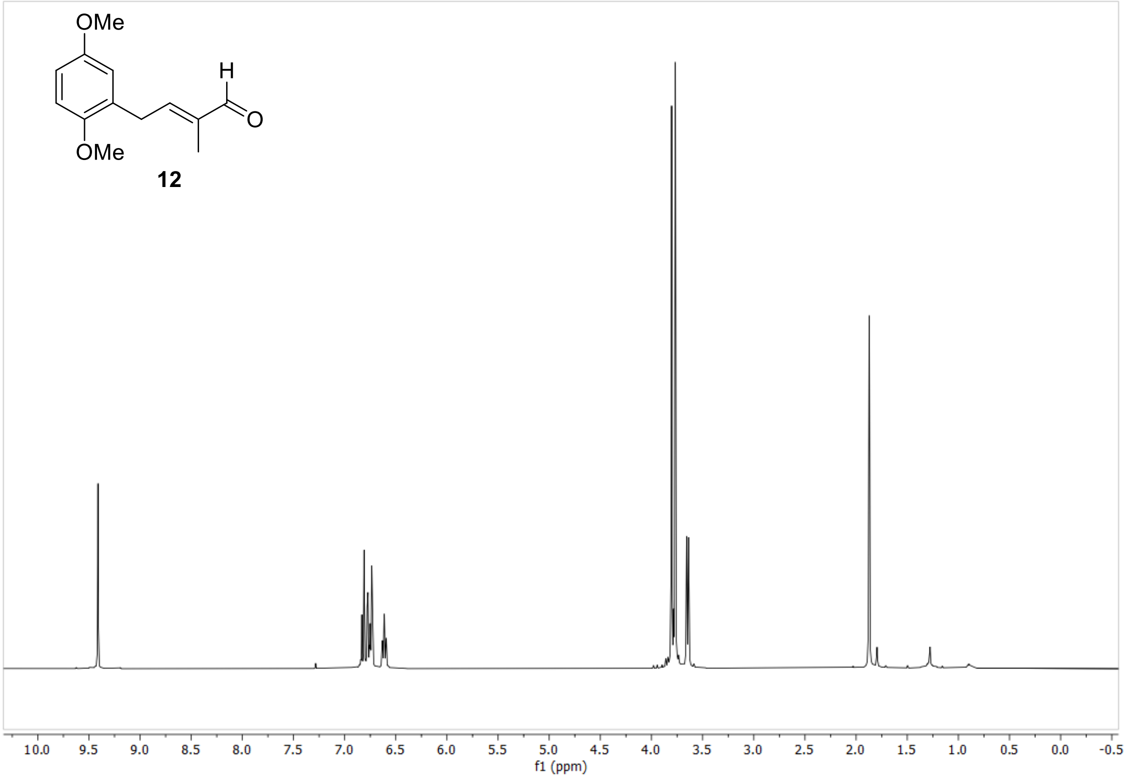
OMe
H
O
OMe
12
10.0
9.5
9.0
8.5
8.0
7.5
7.0
6.5
6.0
5.5
5.0
4.5
4.0
3.5
3.0
2.5
2.0
1.5
1.0
0.5
0.0
-0.5
f1 (ppm)

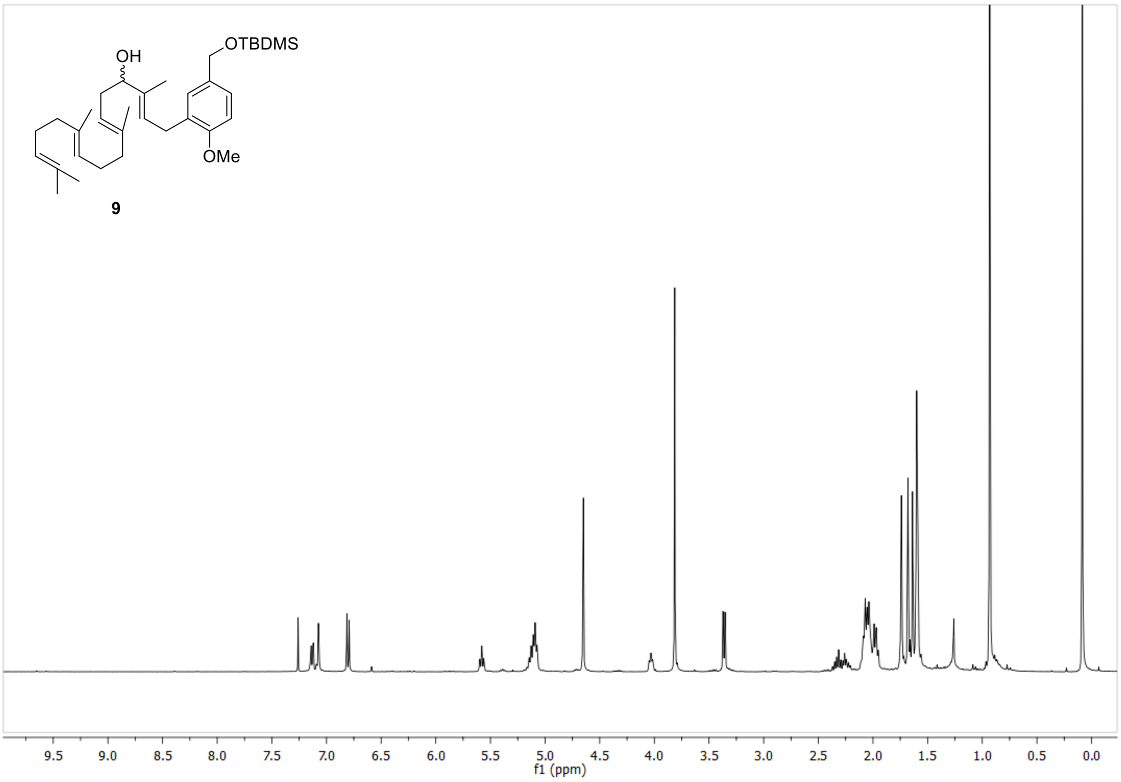
OTBDMS
OH
OMe
9
9.5 9.0 8.5 8.0 7.5 7.0 6.5 6.0 5.5 5.0 4.5 4.0 3.5 3.0 2.5 2.0 1.5 1.0 0.5 0.0
f1 (ppm)

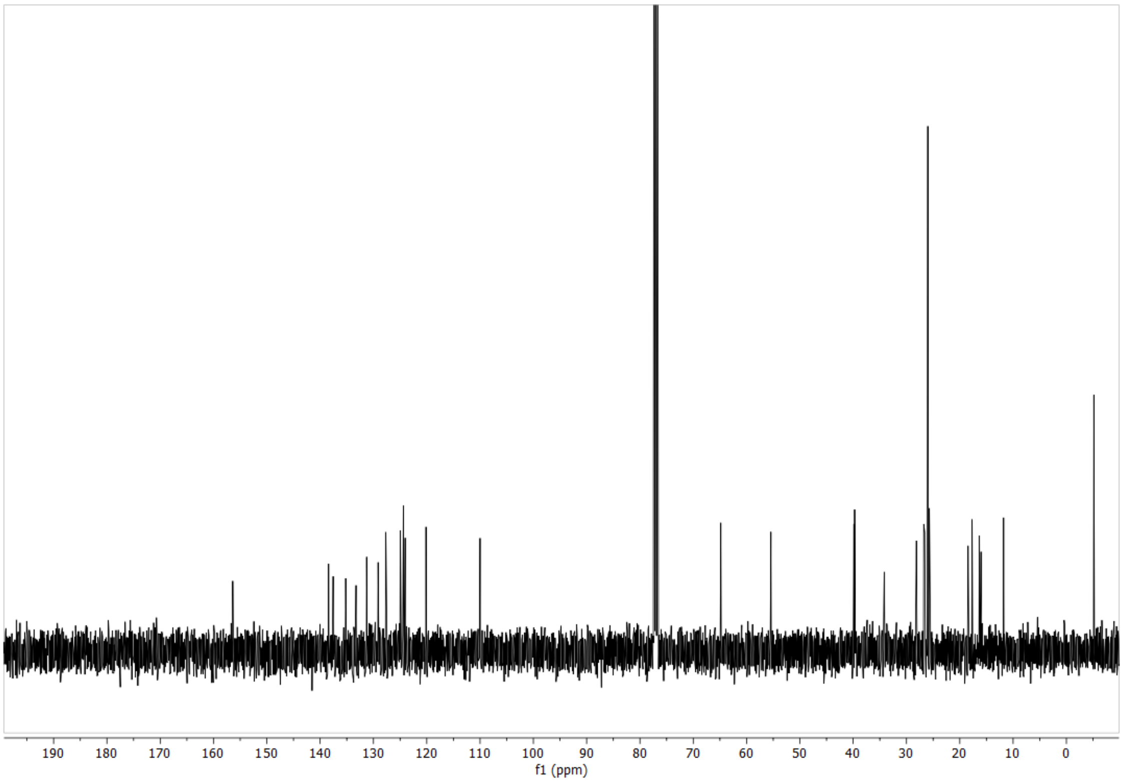
190 180 170 160 150 140 130 120 110 100 90 80 70 60 50 40 30 20 10 0
f1 (ppm)

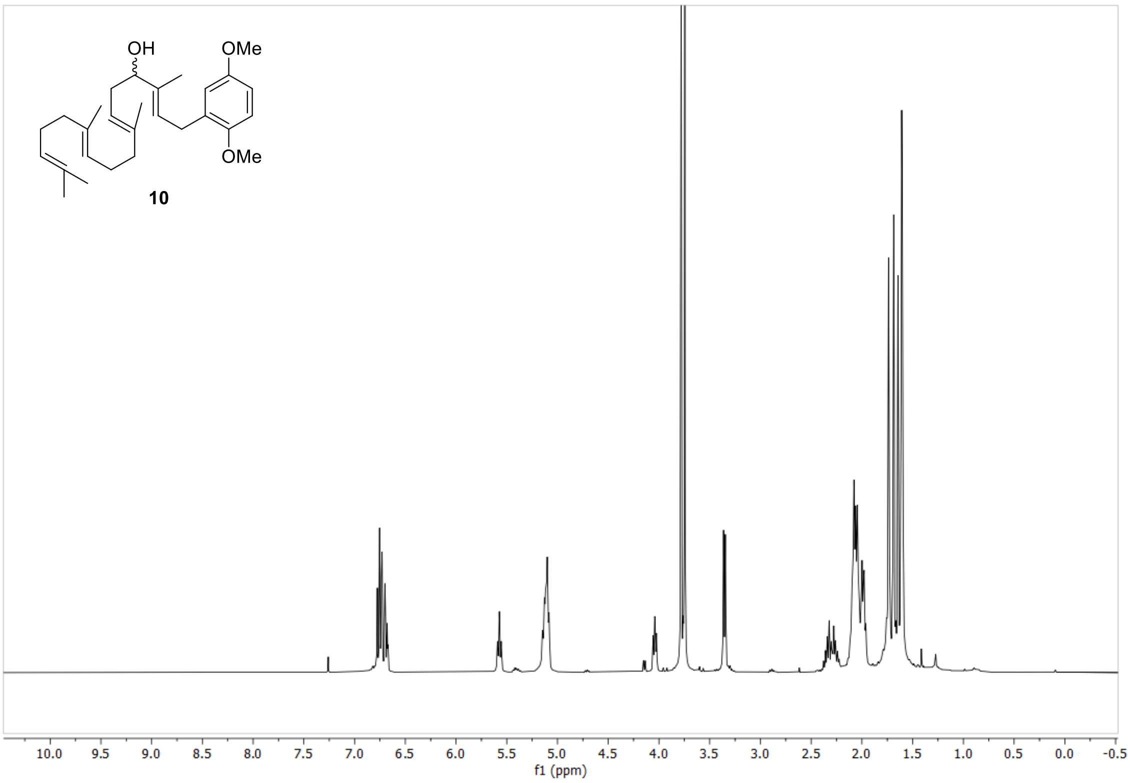
OH
OMe
OMe
10
10.0 9.5 9.0 8.5 8.0 7.5 7.0 6.5 6.0 5.5 5.0 4.5 4.0 3.5 3.0 2.5 2.0 1.5 1.0 0.5 0.0 -0.5
f1 (ppm)

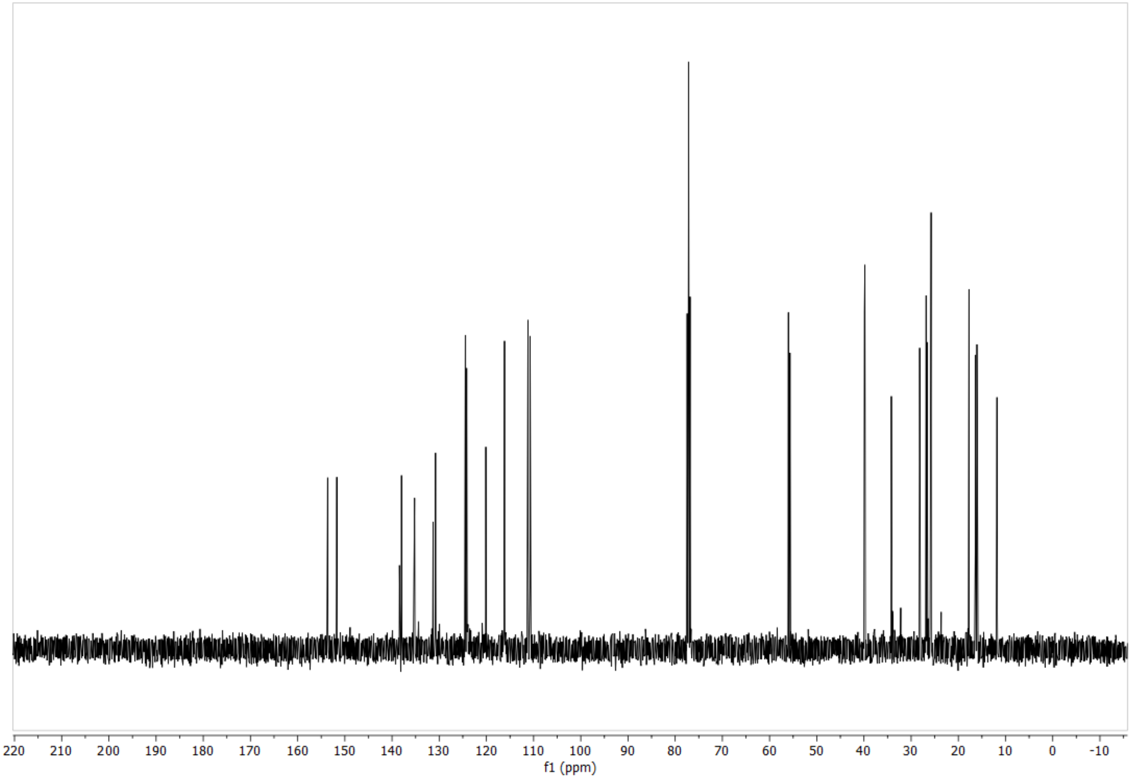
220 210 200 190 180 170 160 150 140 130 120 110 100 90 80 70 60 50 40 30 20 10 0 -10
f1 (ppm)

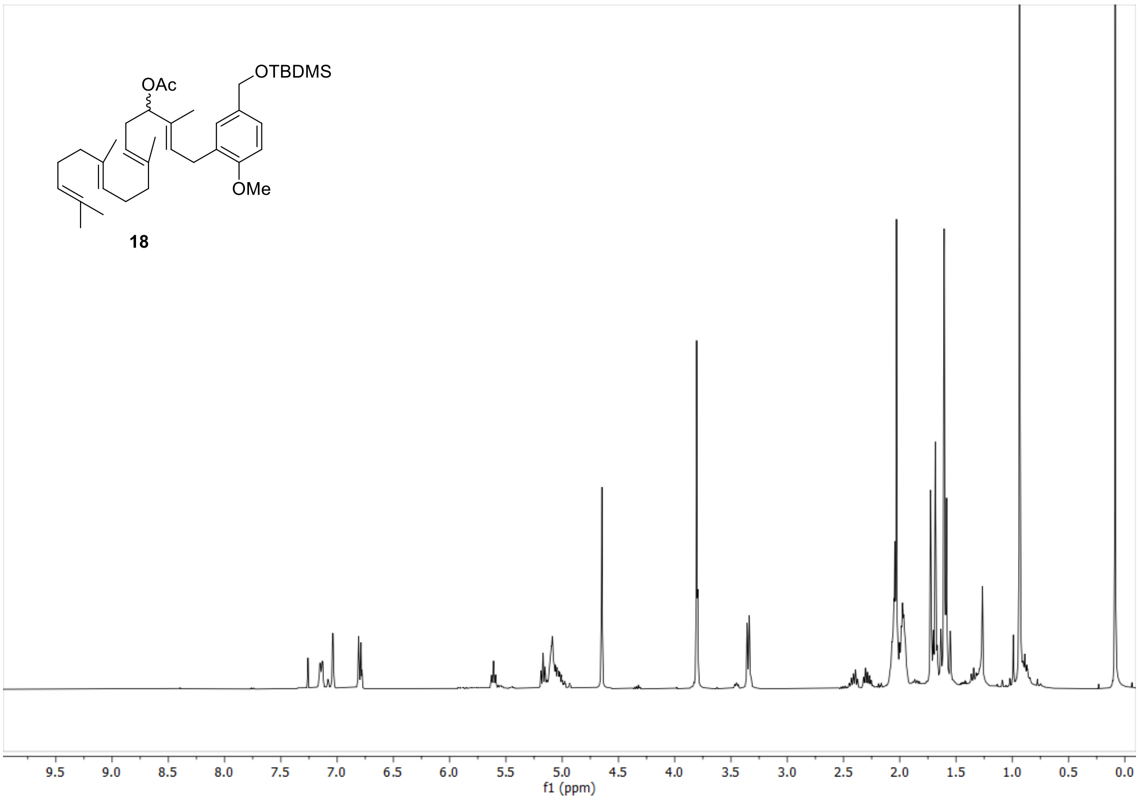
OTBDMS
OAc
OMe
18
9.5 9.0 8.5 8.0 7.5 7.0 6.5 6.0 5.5 5.0 4.5 4.0 3.5 3.0 2.5 2.0 1.5 1.0 0.5 0.0
f1 (ppm)

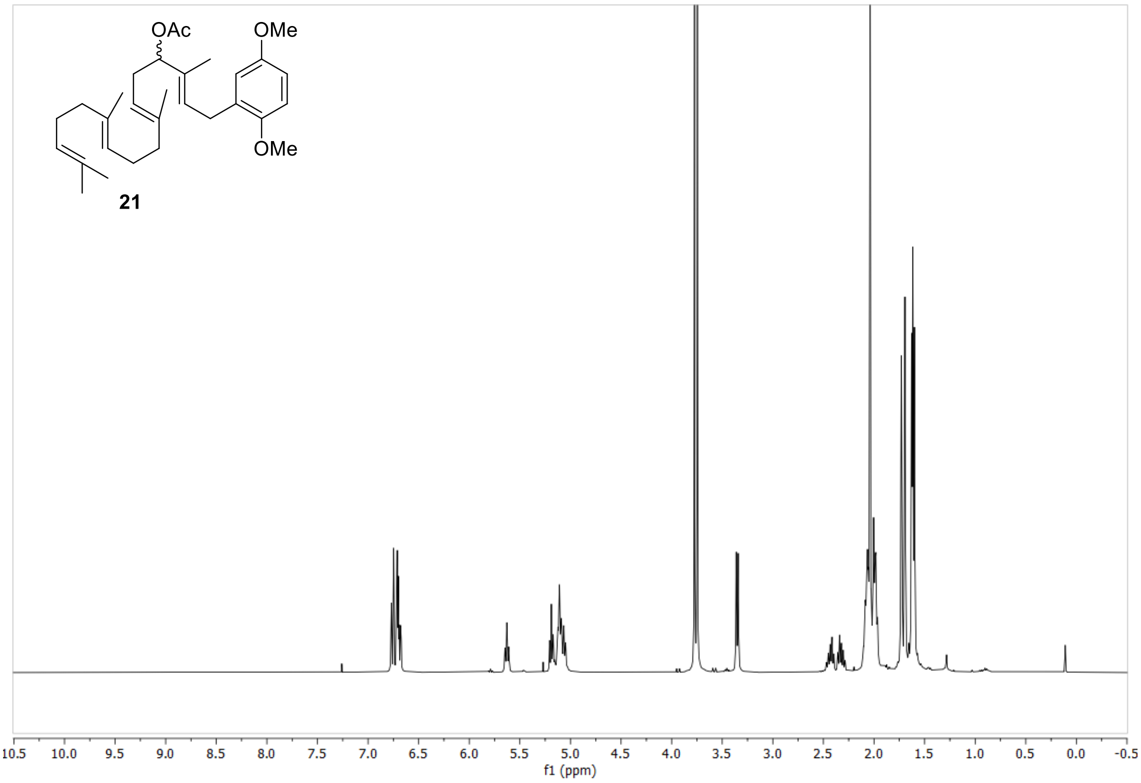
OAc
OMe
OMe
21
10.5 10.0 9.5 9.0 8.5 8.0 7.5 7.0 6.5 6.0 5.5 5.0 4.5 4.0 3.5 3.0 2.5 2.0 1.5 1.0 0.5 0.0 -0.5
f1 (ppm)

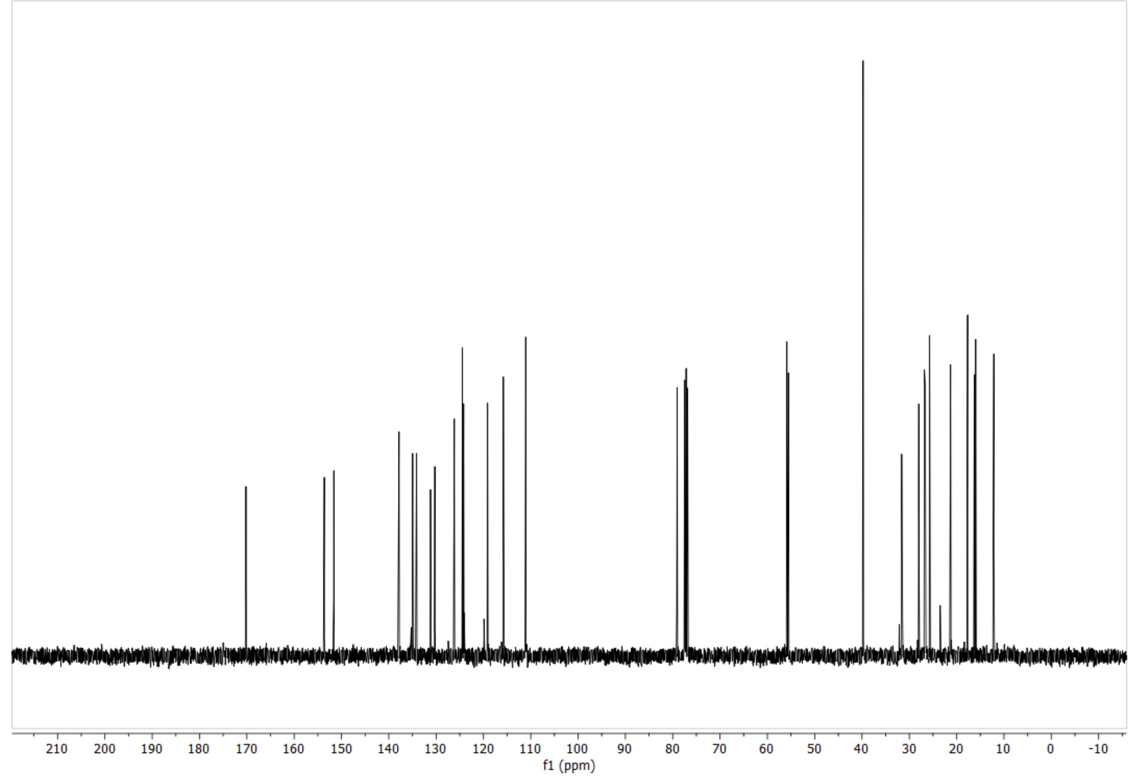
210 200 190 180 170 160 150 140 130 120 110 100 90 80 70 60 50 40 30 20 10 0 -10
f1 (ppm)

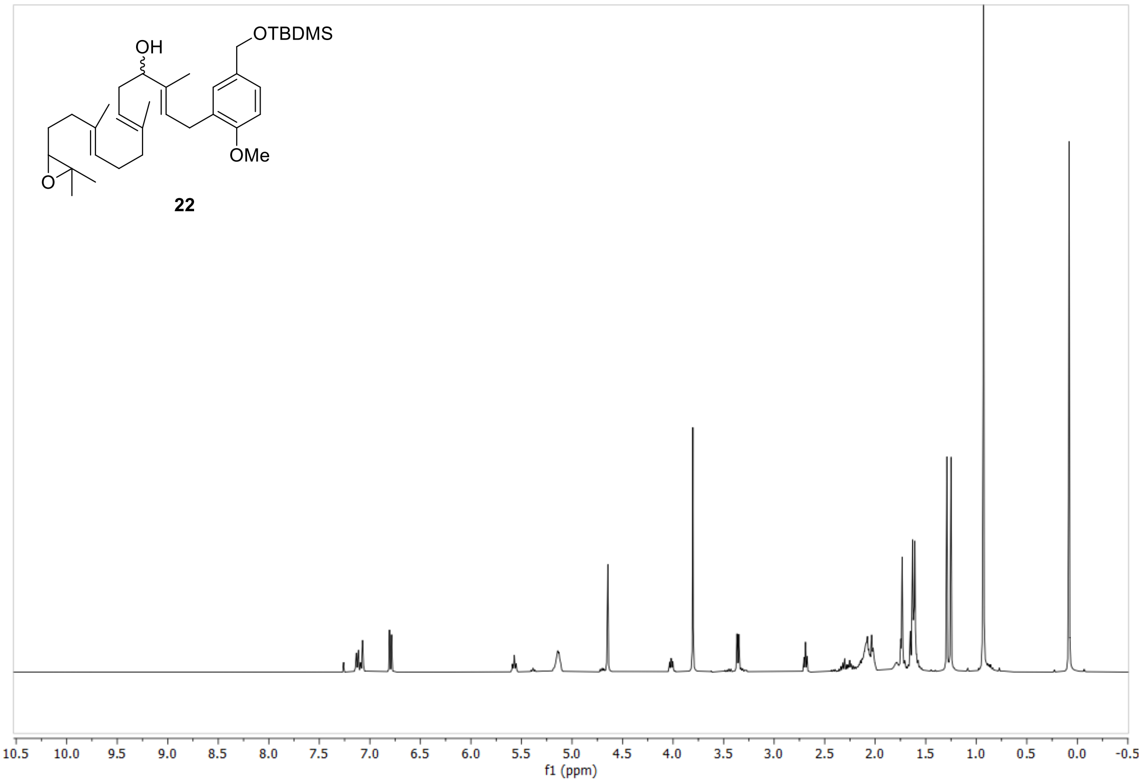
OTBDMS
OH
OMe
O
22
10.5 10.0 9.5 9.0 8.5 8.0 7.5 7.0 6.5 6.0 5.5 5.0 4.5 4.0 3.5 3.0 2.5 2.0 1.5 1.0 0.5 0.0 -0.5
f1 (ppm)

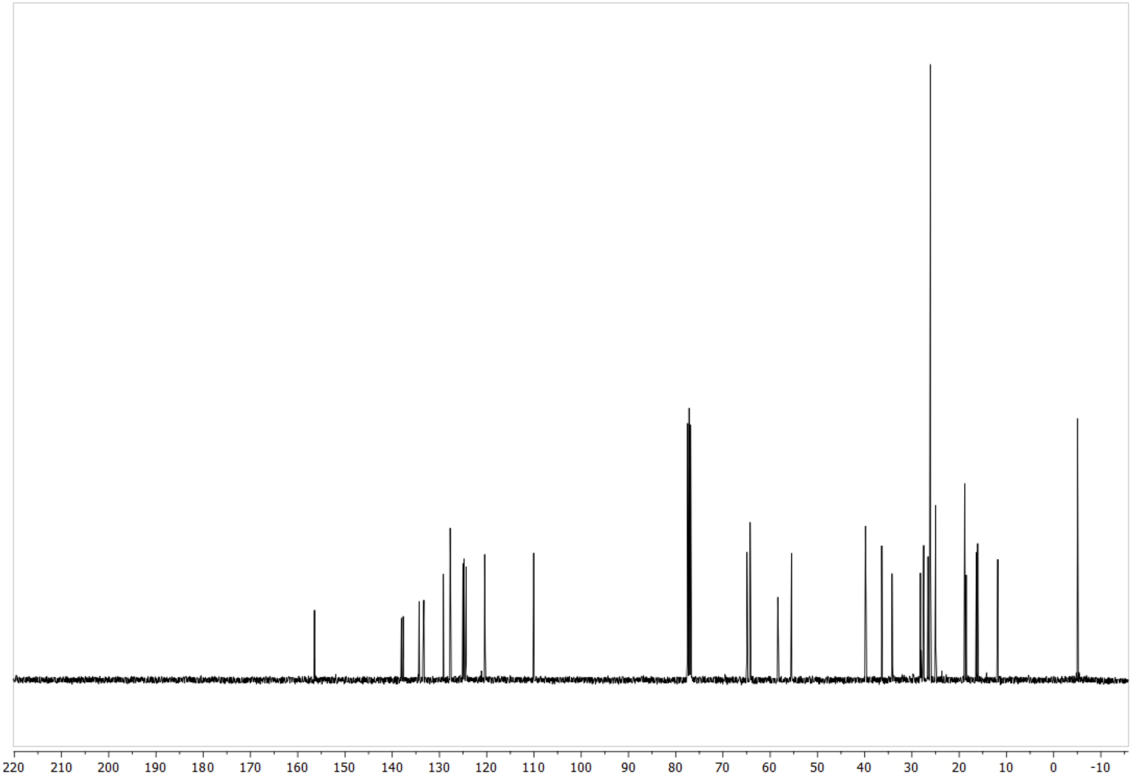
220 210 200 190 180 170 160 150 140 130 120 110 100 90 80 70 60 50 40 30 20 10 0 -10

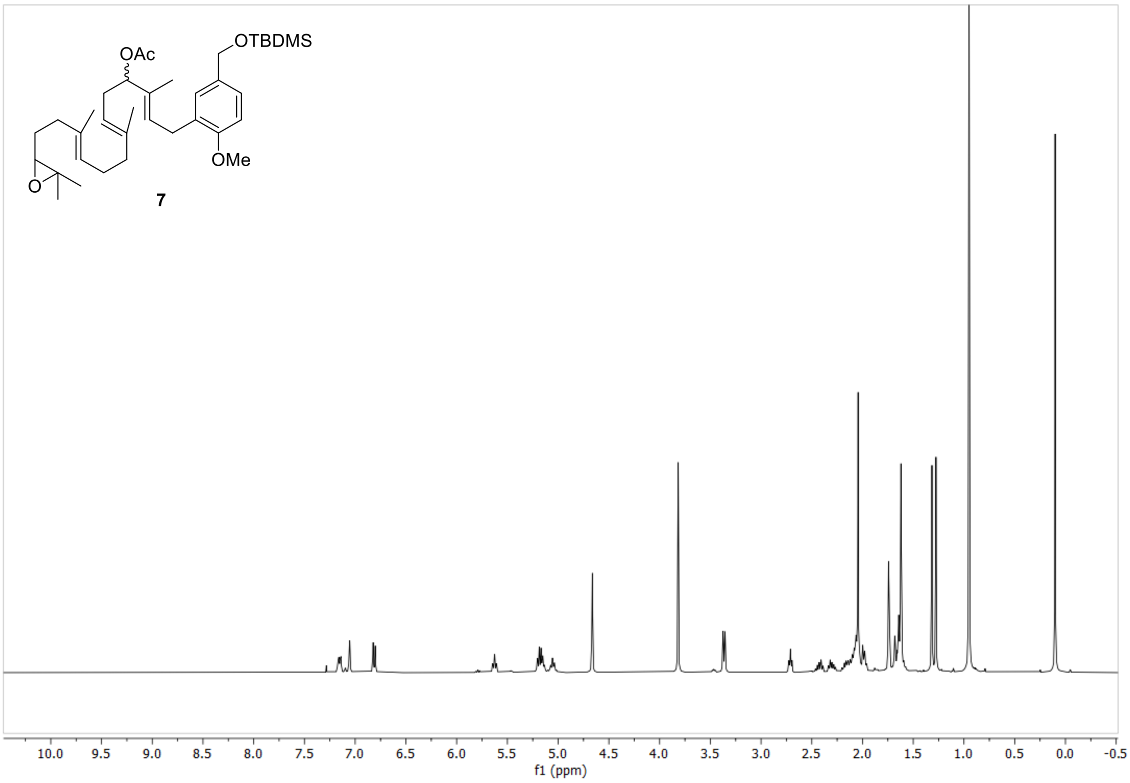
OTBDMS
OAc
O
OMe
7
10.0 9.5 9.0 8.5 8.0 7.5 7.0 6.5 6.0 5.5 5.0 4.5 4.0 3.5 3.0 2.5 2.0 1.5 1.0 0.5 0.0 -0.5
f1 (ppm)

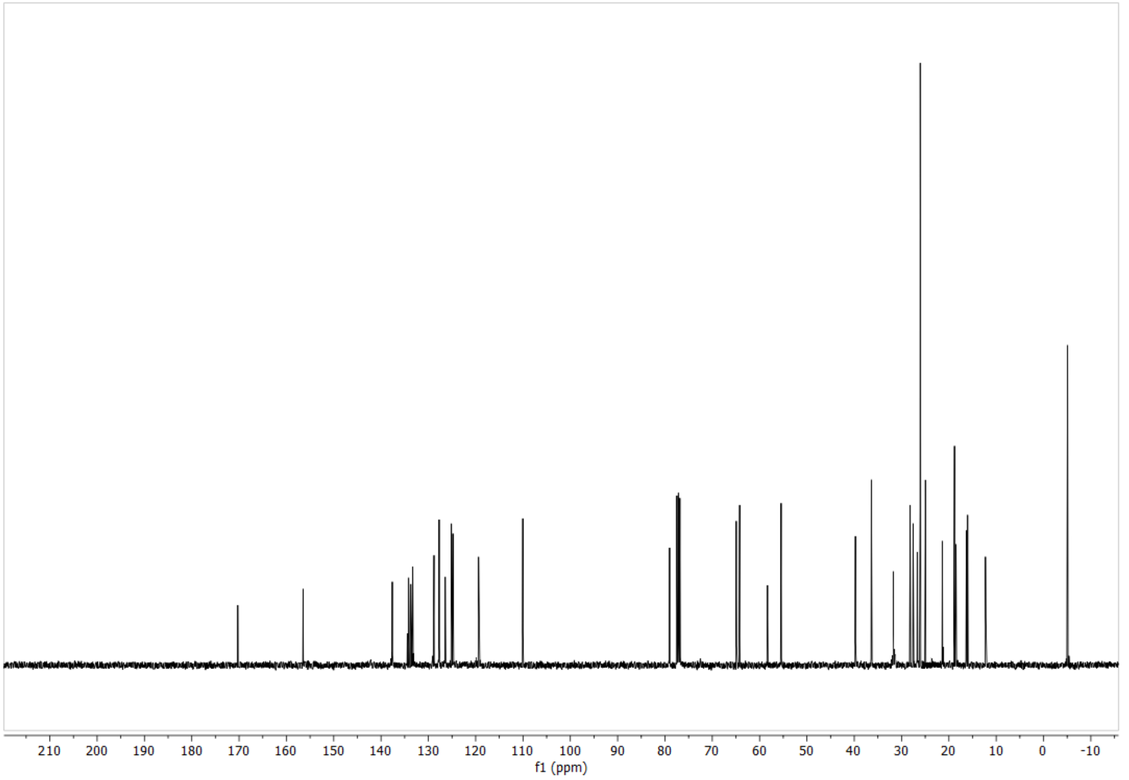
210 200 190 180 170 160 150 140 130 120 110 100 90 80 70 60 50 40 30 20 10 0 -10
f1 (ppm)

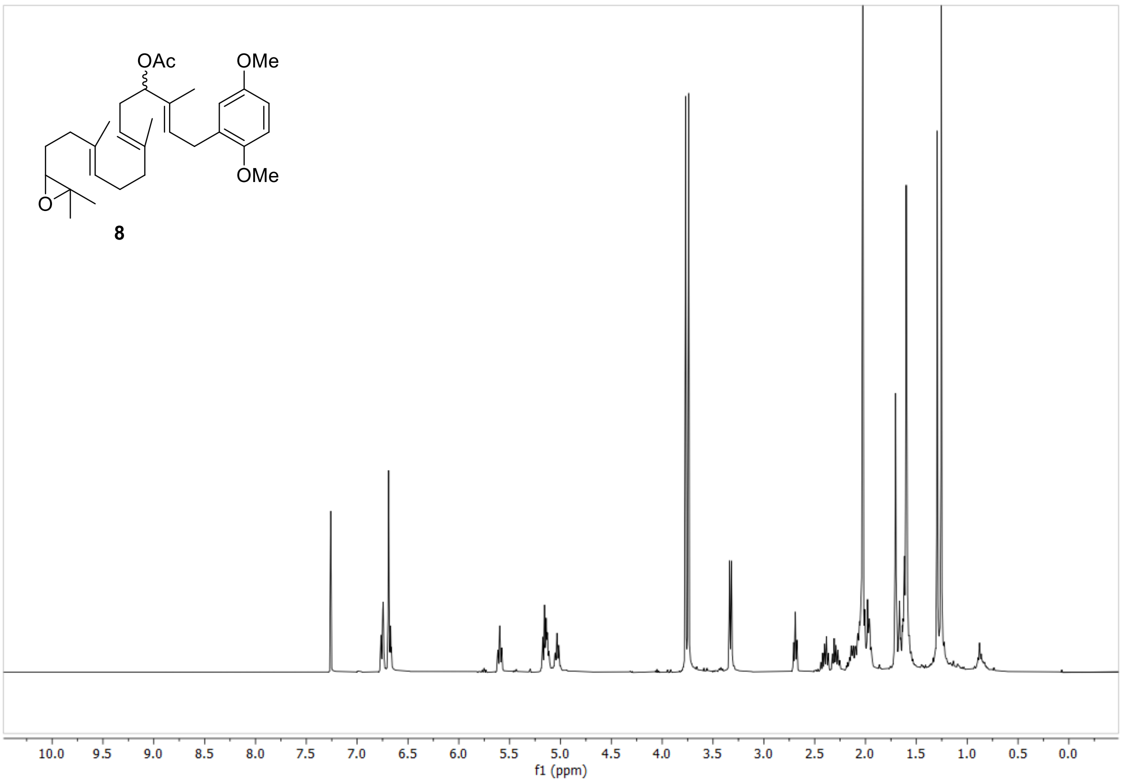
OAc
OMe
OMe
O
8
10.0 9.5 9.0 8.5 8.0 7.5 7.0 6.5 6.0 5.5 5.0 4.5 4.0 3.5 3.0 2.5 2.0 1.5 1.0 0.5 0.0
f1 (ppm)

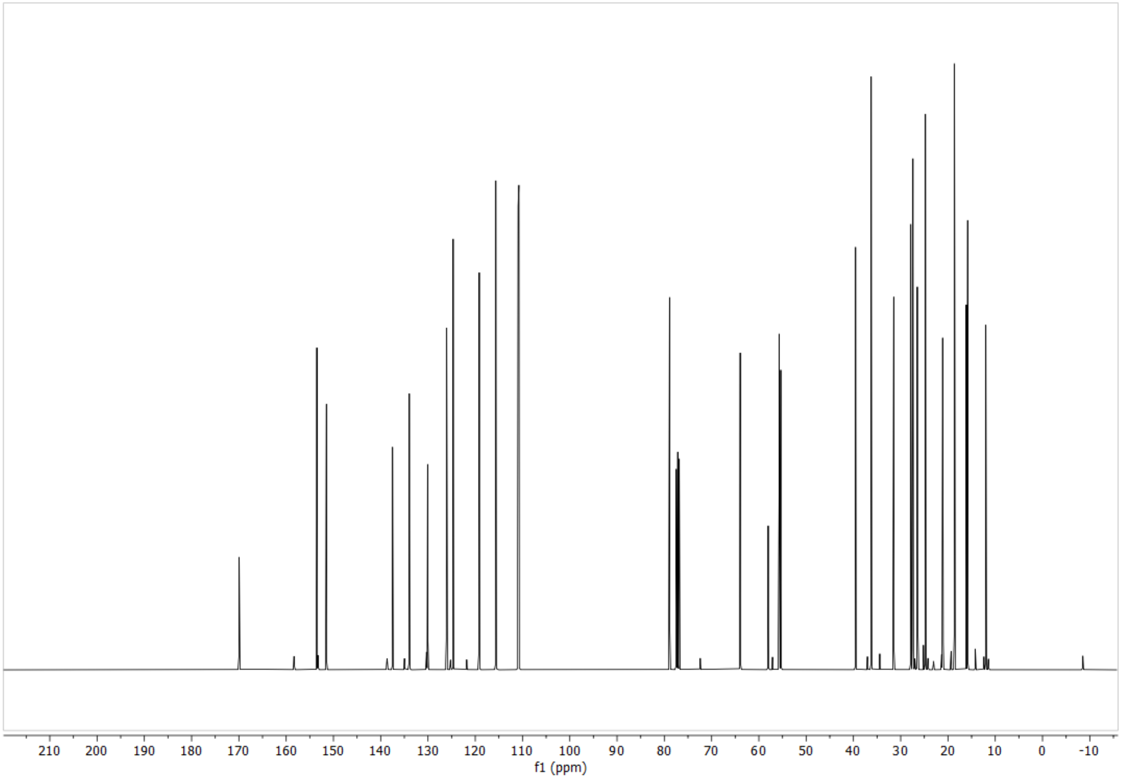
210 200 190 180 170 160 150 140 130 120 110 100 90 80 70 60 50 40 30 20 10 0 -10
f1 (ppm)

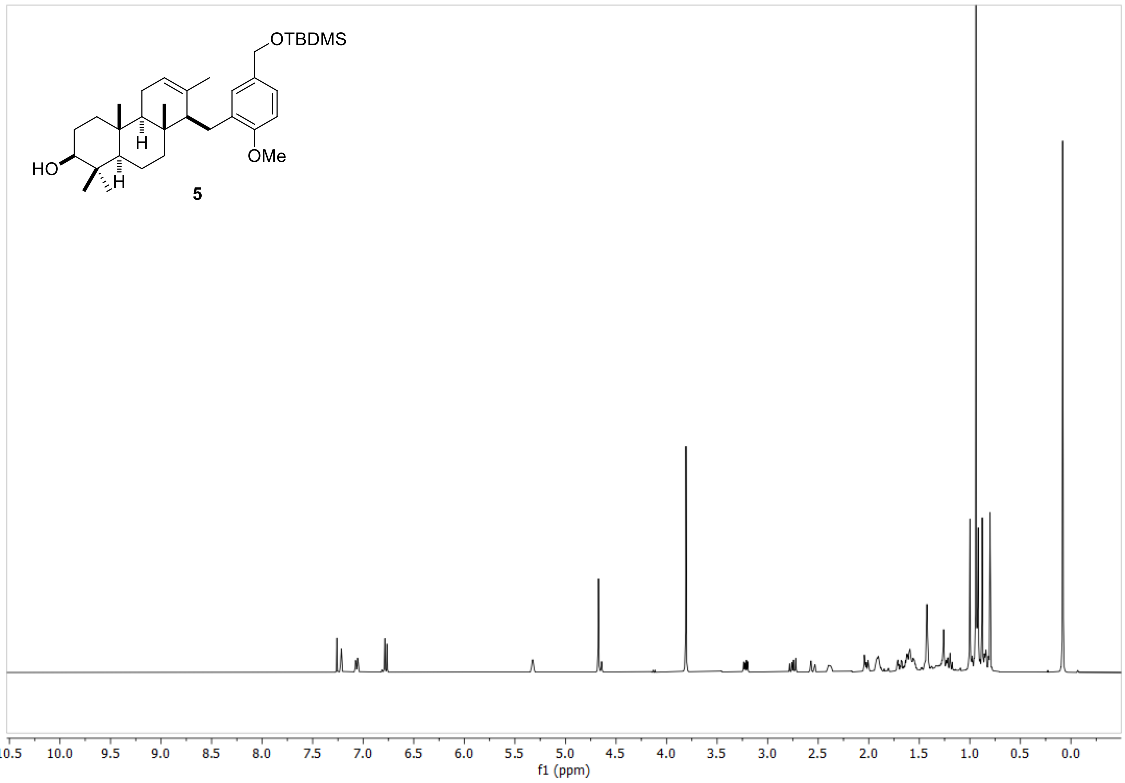
OTBDMS
HO
H
H
OMe
5
10.5 10.0 9.5 9.0 8.5 8.0 7.5 7.0 6.5 6.0 5.5 5.0 4.5 4.0 3.5 3.0 2.5 2.0 1.5 1.0 0.5 0.0
f1 (ppm)

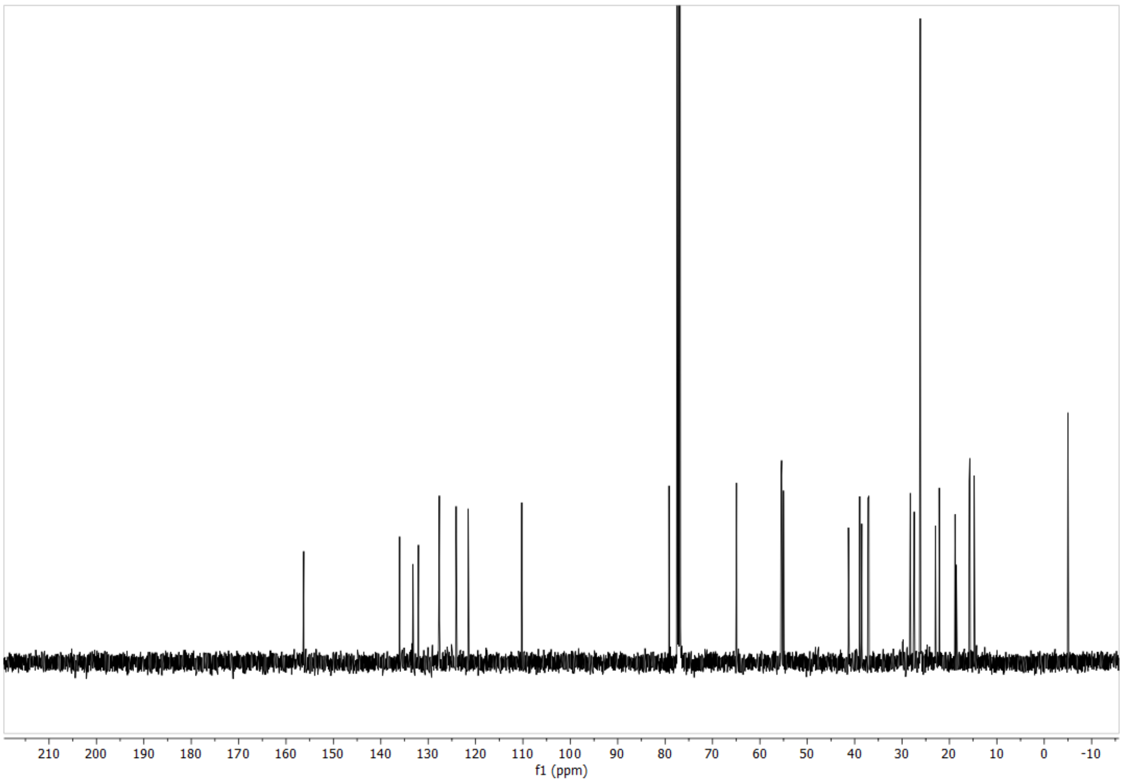
210 200 190 180 170 160 150 140 130 120 110 100 90 80 70 60 50 40 30 20 10 0 -10
f1 (ppm)

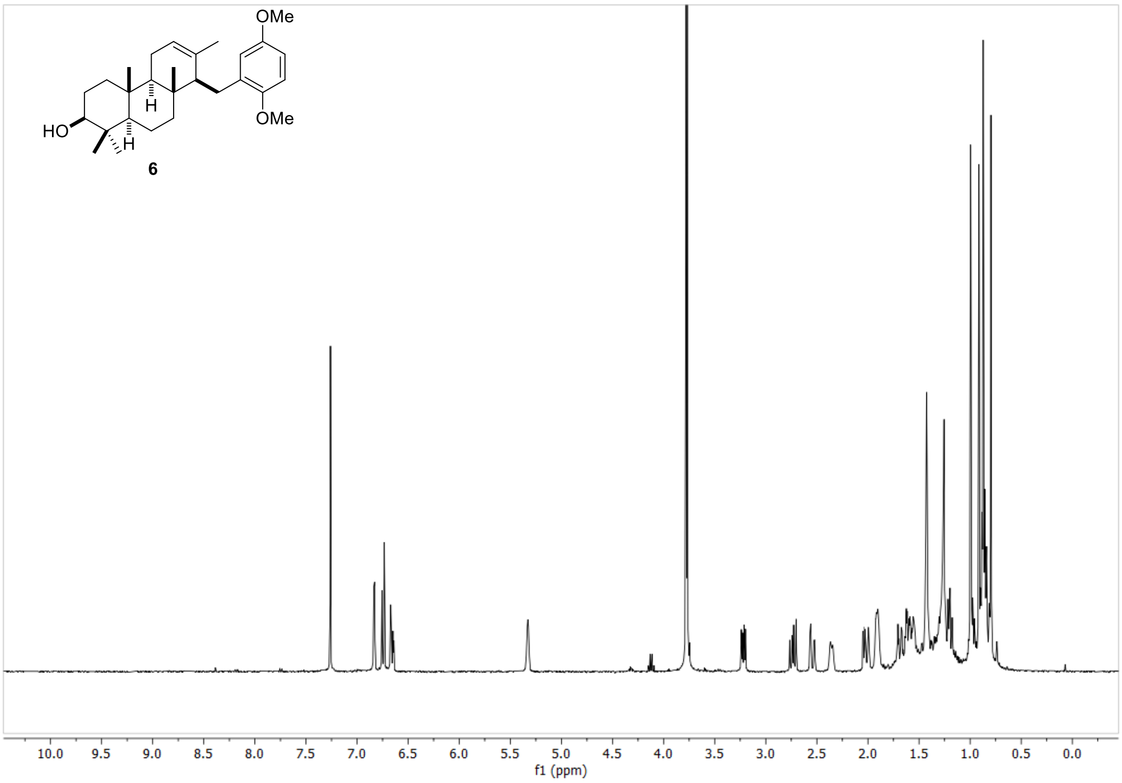
OMe
OMe
HO
H
H
6
10.0 9.5 9.0 8.5 8.0 7.5 7.0 6.5 6.0 5.5 5.0 4.5 4.0 3.5 3.0 2.5 2.0 1.5 1.0 0.5 0.0
f1 (ppm)

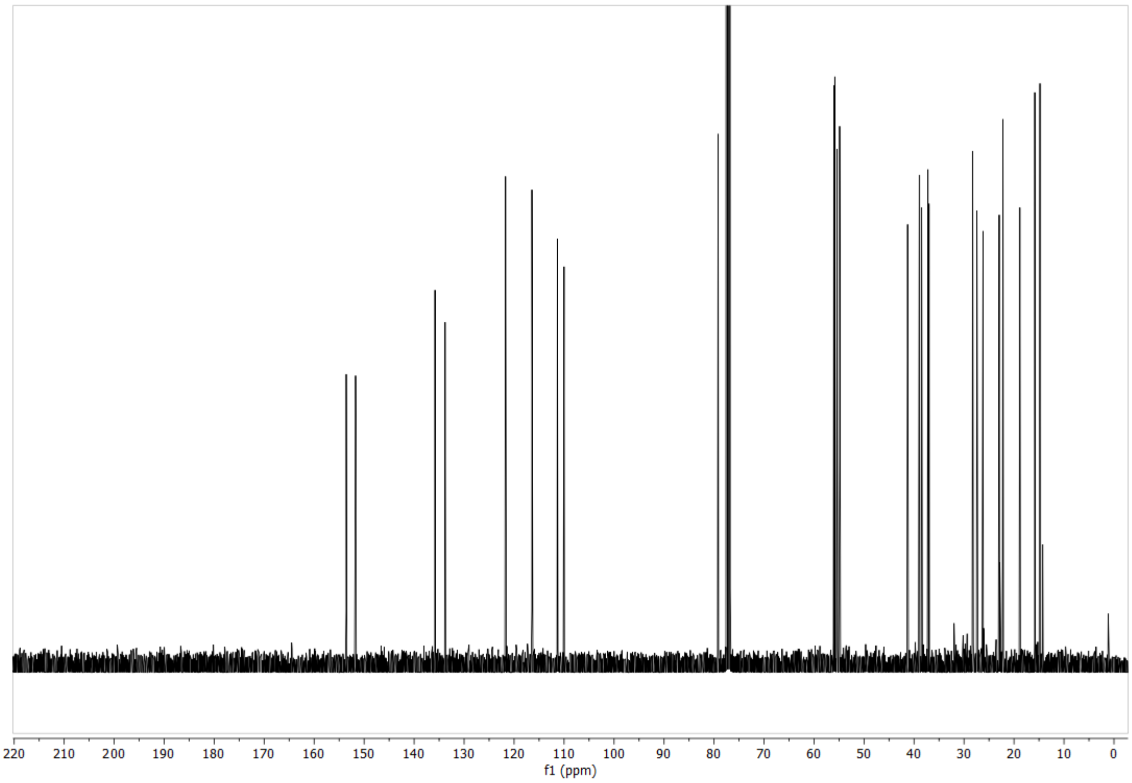
220 210 200 190 180 170 160 150 140 130 120 110 100 90 80 70 60 50 40 30 20 10 0
f1 (ppm)

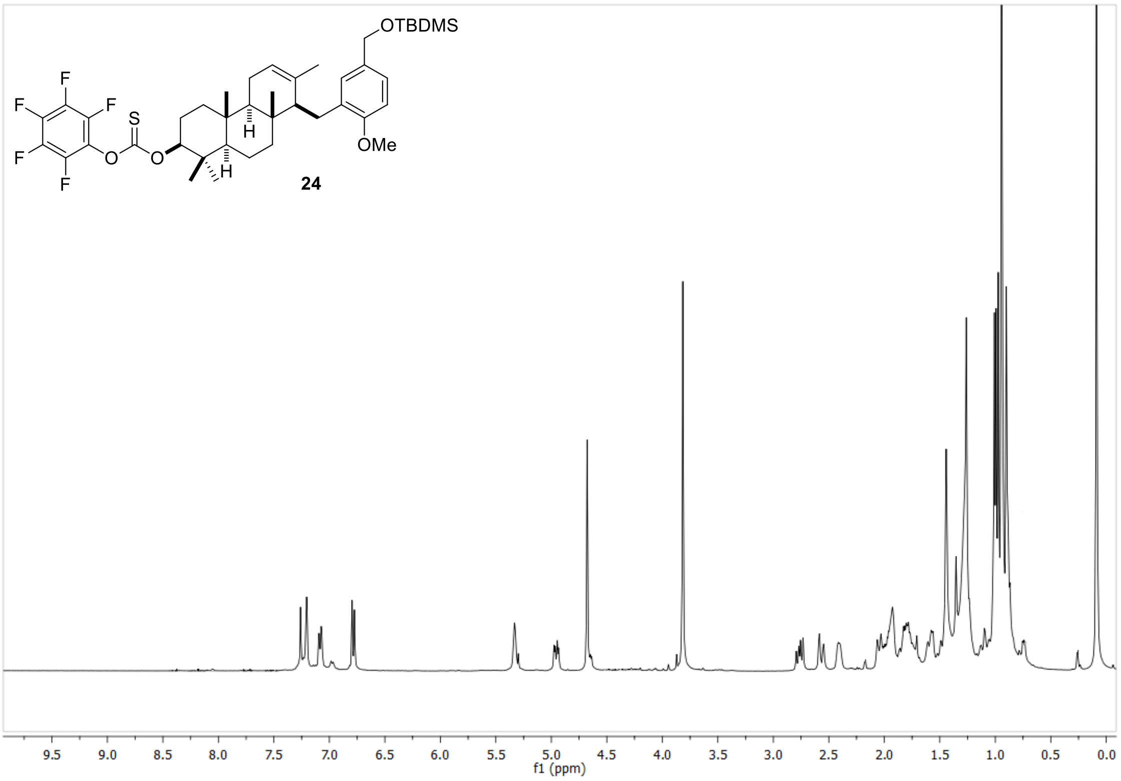
OTBDMS
F
F
F
F
F
S
O
O
H
H
OMe
24
9.5 9.0 8.5 8.0 7.5 7.0 6.5 6.0 5.5 5.0 4.5 4.0 3.5 3.0 2.5 2.0 1.5 1.0 0.5 0.0
f1 (ppm)

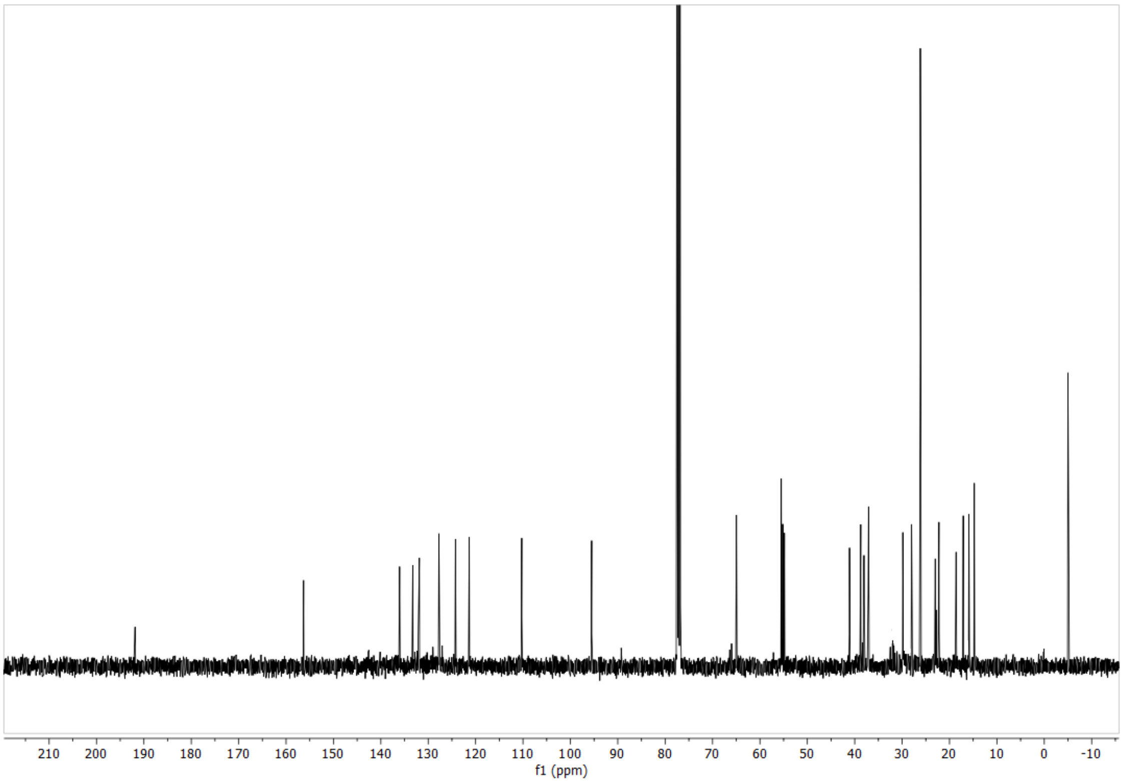
210 200 190 180 170 160 150 140 130 120 110 100 90 80 70 60 50 40 30 20 10 0 -10
f1 (ppm)

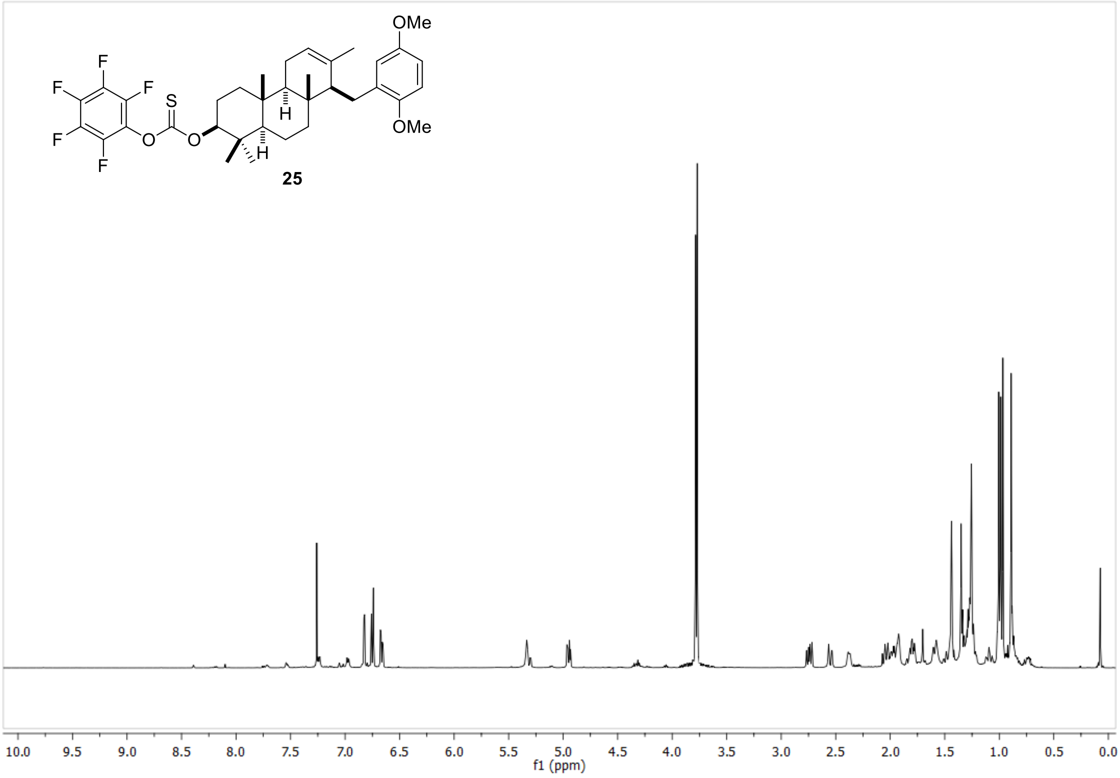
OMe
F
F
F
F
F
S
O
O
H
H
OMe
25
10.0 9.5 9.0 8.5 8.0 7.5 7.0 6.5 6.0 5.5 5.0 4.5 4.0 3.5 3.0 2.5 2.0 1.5 1.0 0.5 0.0
f1 (ppm)

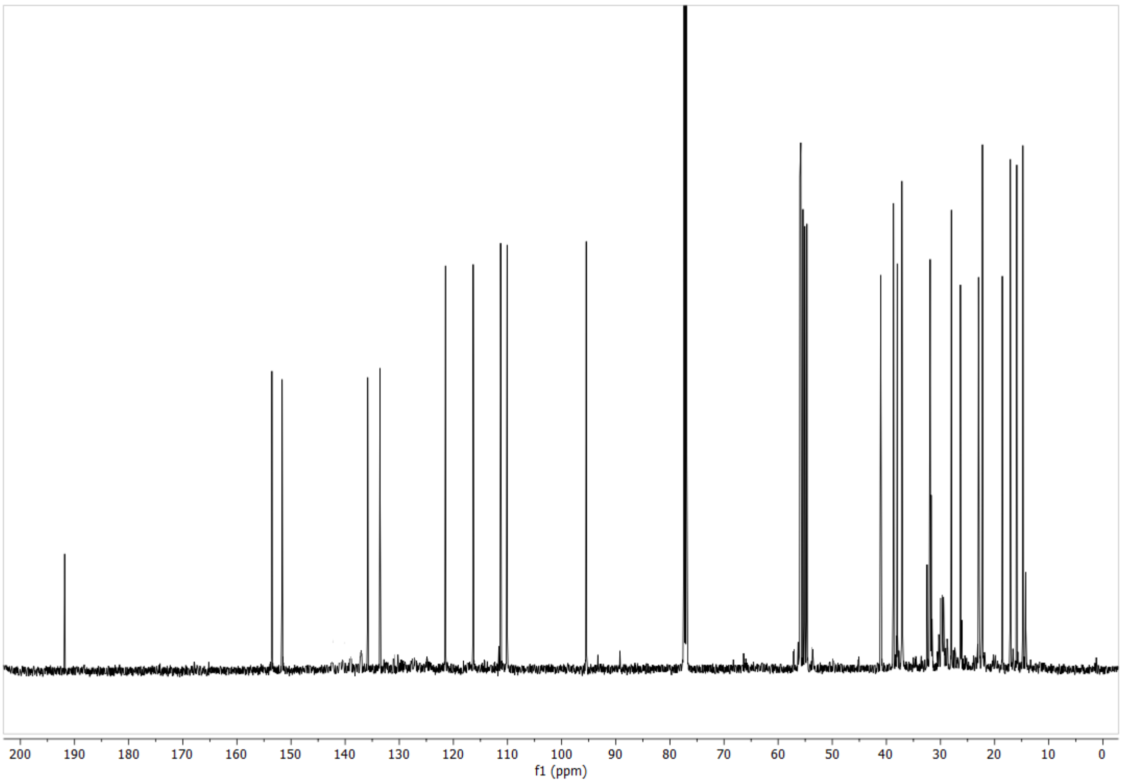
200 190 180 170 160 150 140 130 120 110 100 90 80 70 60 50 40 30 20 10 0
f1 (ppm)

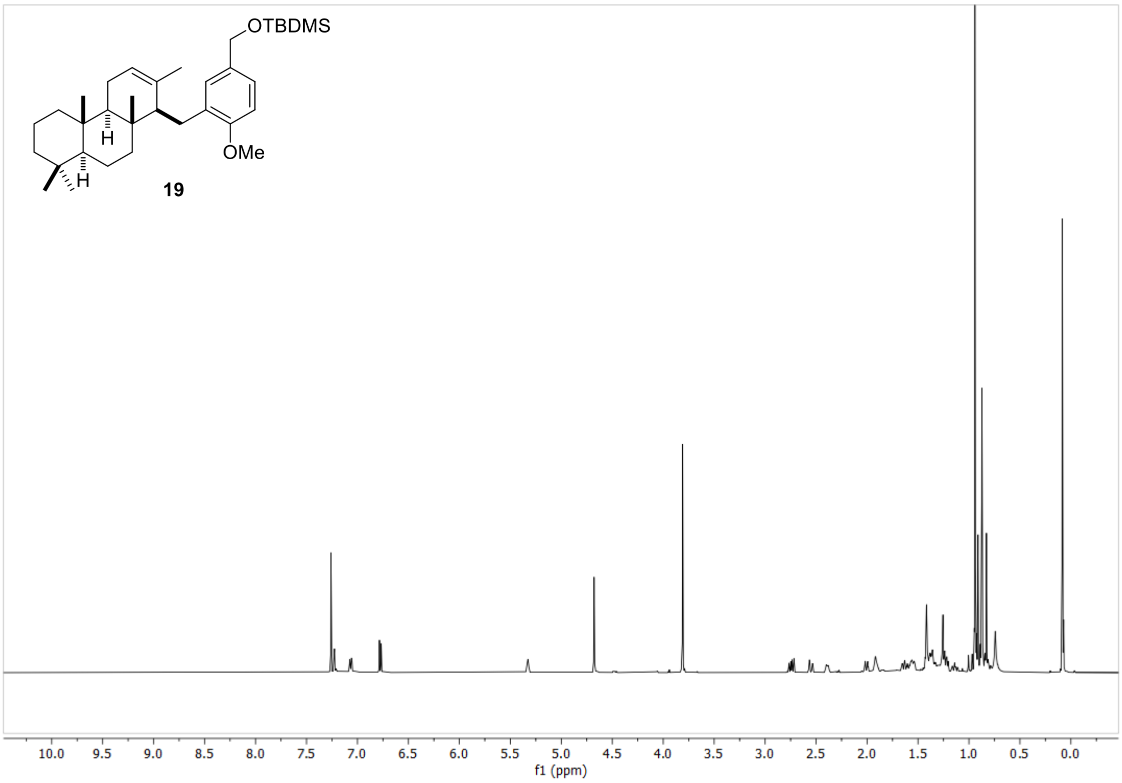
OTBDMS
H
H
OMe
19
10.0 9.5 9.0 8.5 8.0 7.5 7.0 6.5 6.0 5.5 5.0 4.5 4.0 3.5 3.0 2.5 2.0 1.5 1.0 0.5 0.0
f1 (ppm)

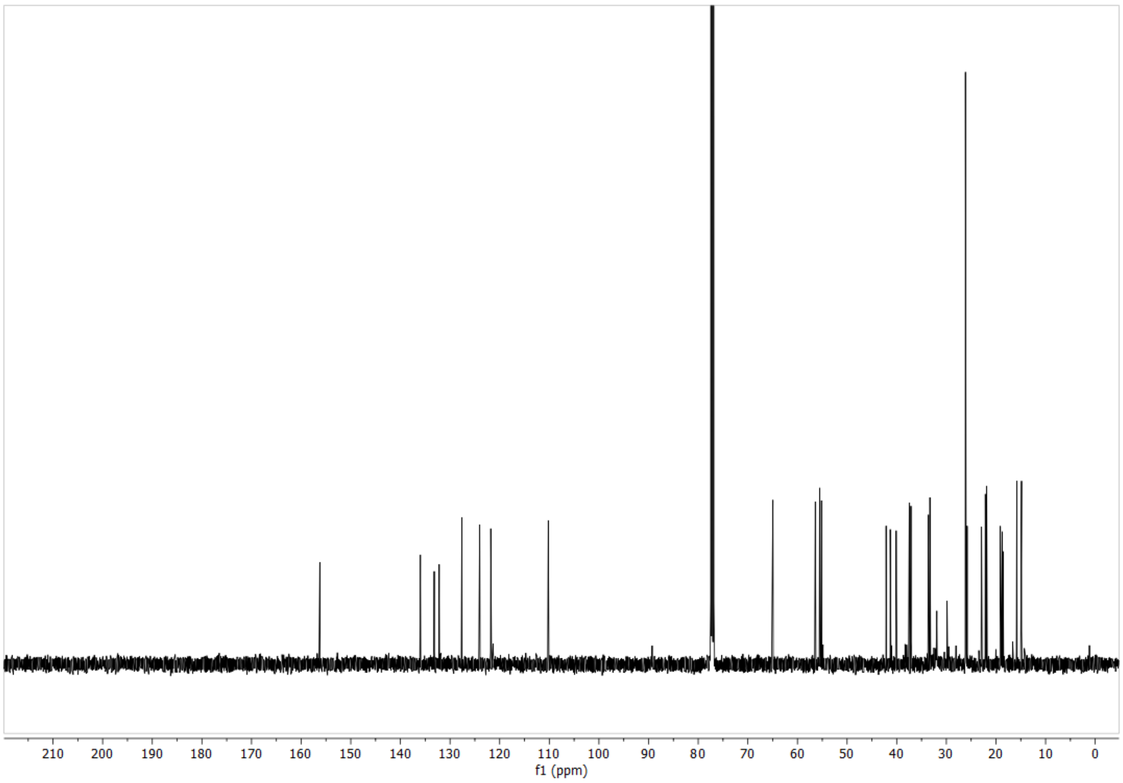
210 200 190 180 170 160 150 140 130 120 110 100 90 80 70 60 50 40 30 20 10 0
f1 (ppm)

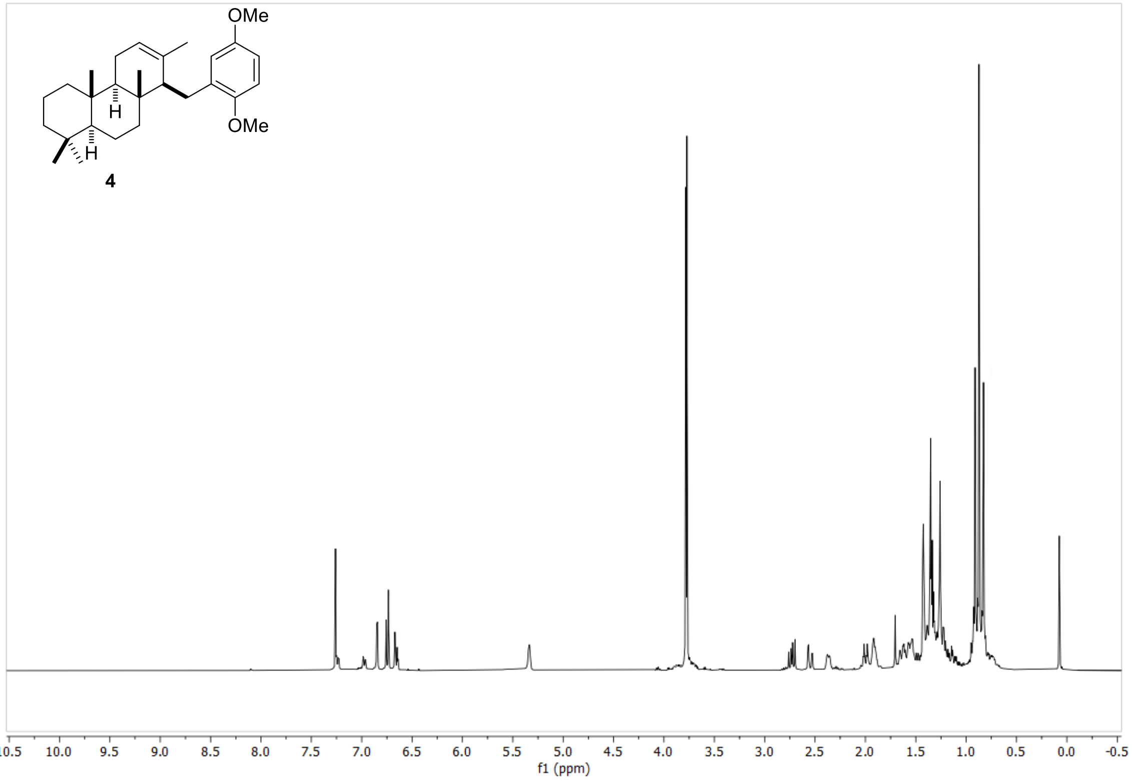
OMe
OMe
H
H
4
10.5 10.0 9.5 9.0 8.5 8.0 7.5 7.0 6.5 6.0 5.5 5.0 4.5 4.0 3.5 3.0 2.5 2.0 1.5 1.0 0.5 0.0 -0.5
f1 (ppm)

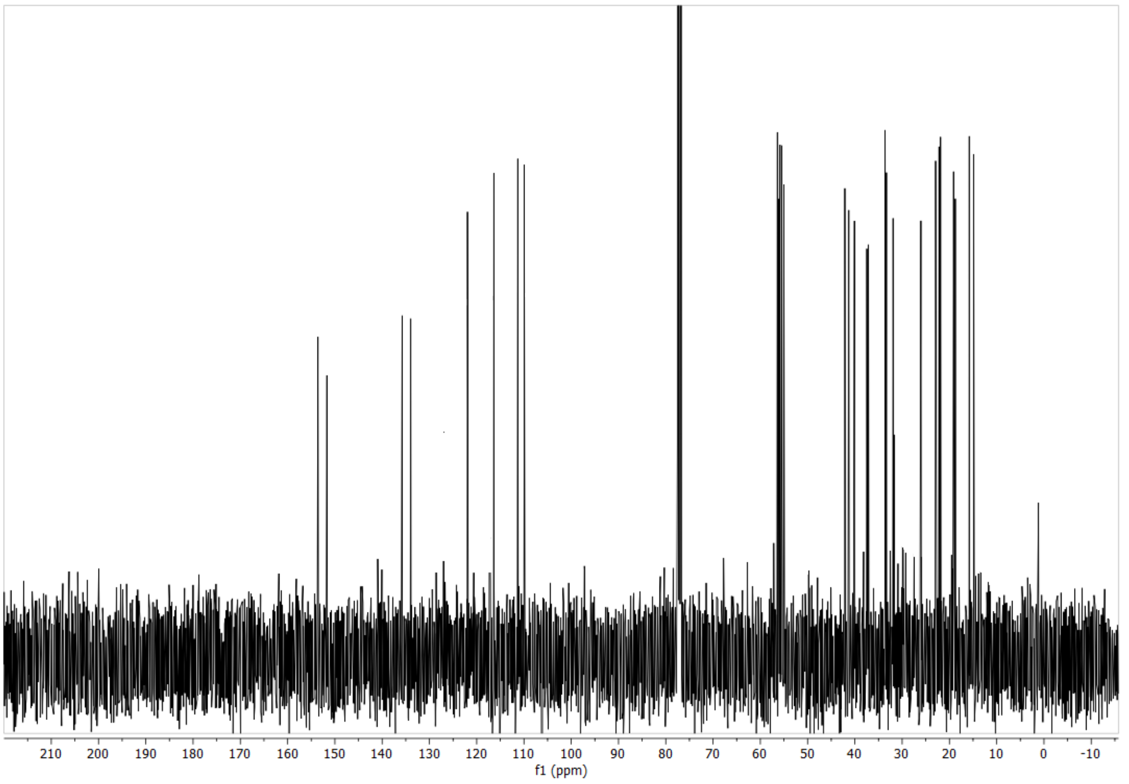
210 200 190 180 170 160 150 140 130 120 110 100 90 80 70 60 50 40 30 20 10 0 -10
f1 (ppm)

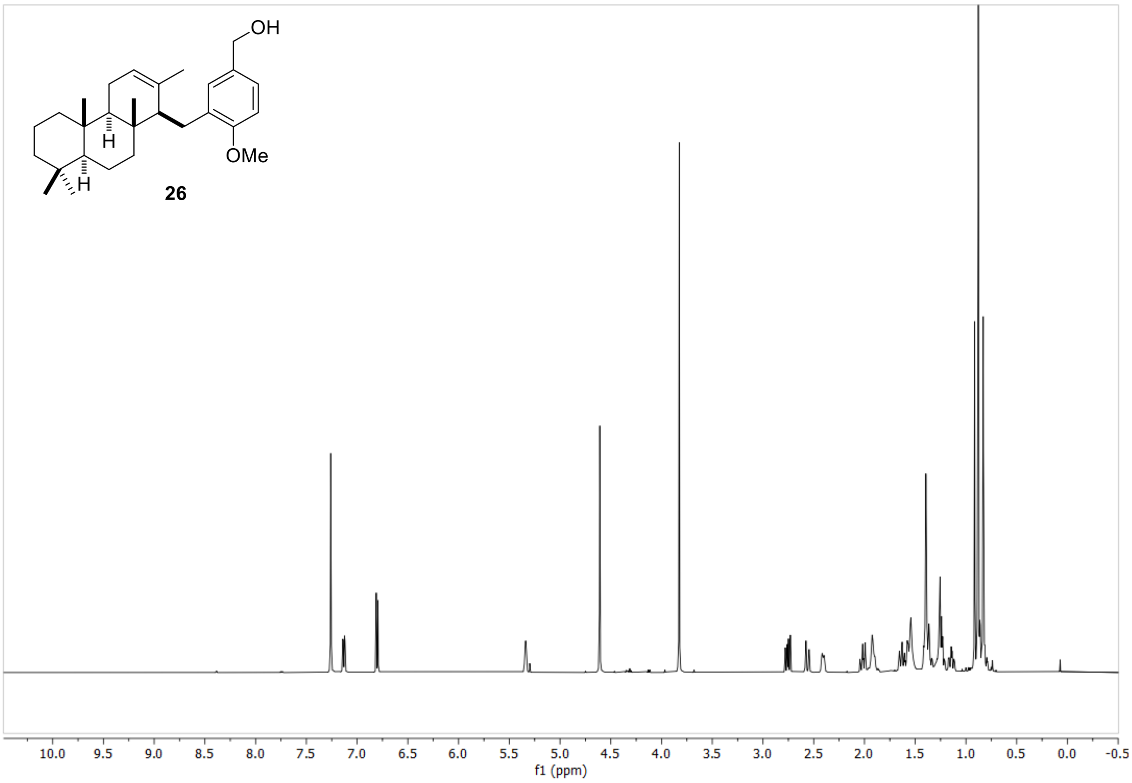
OH
OMe
H
H
26
10.0 9.5 9.0 8.5 8.0 7.5 7.0 6.5 6.0 5.5 5.0 4.5 4.0 3.5 3.0 2.5 2.0 1.5 1.0 0.5 0.0 -0.5
f1 (ppm)

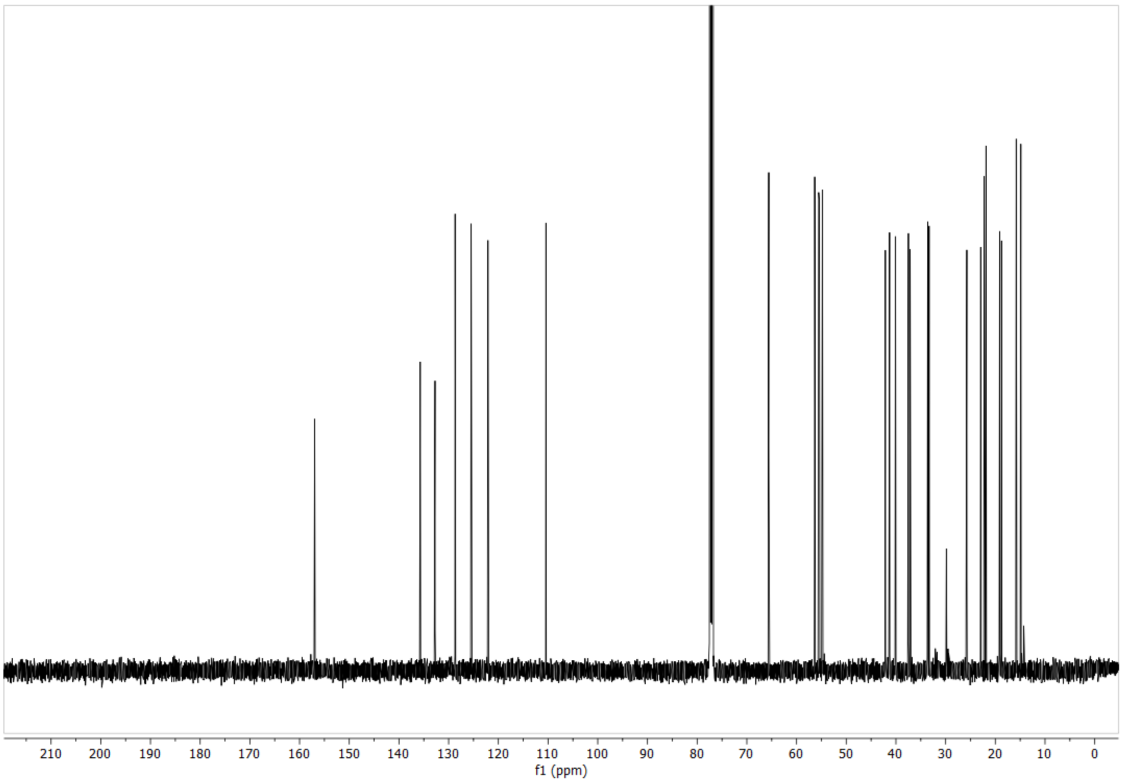
210 200 190 180 170 160 150 140 130 120 110 100 90 80 70 60 50 40 30 20 10 0
f1 (ppm)

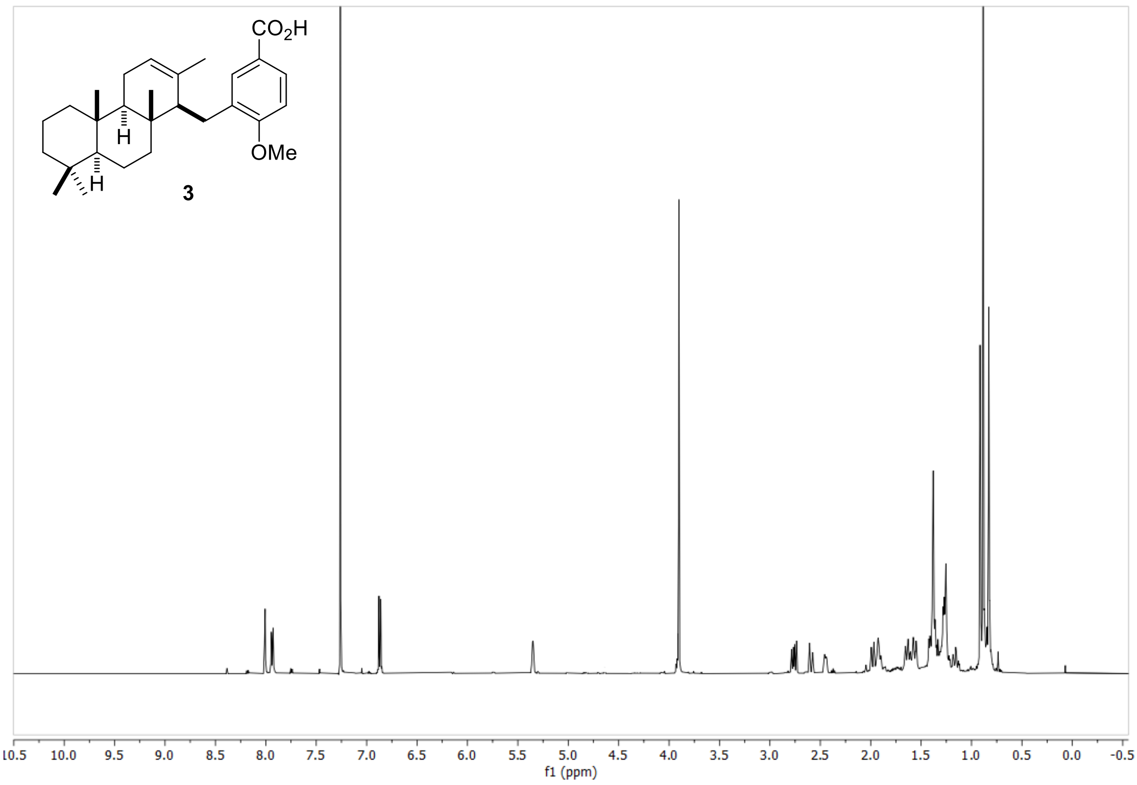

CO2H
H
H
OMe
3
10.5
10.0
9.5
9.0
8.5
8.0
7.5
7.0
6.5
6.0
5.5
5.0
4.5
4.0
3.5
3.0
2.5
2.0
1.5
1.0
0.5
0.0
-0.5
f1 (ppm)

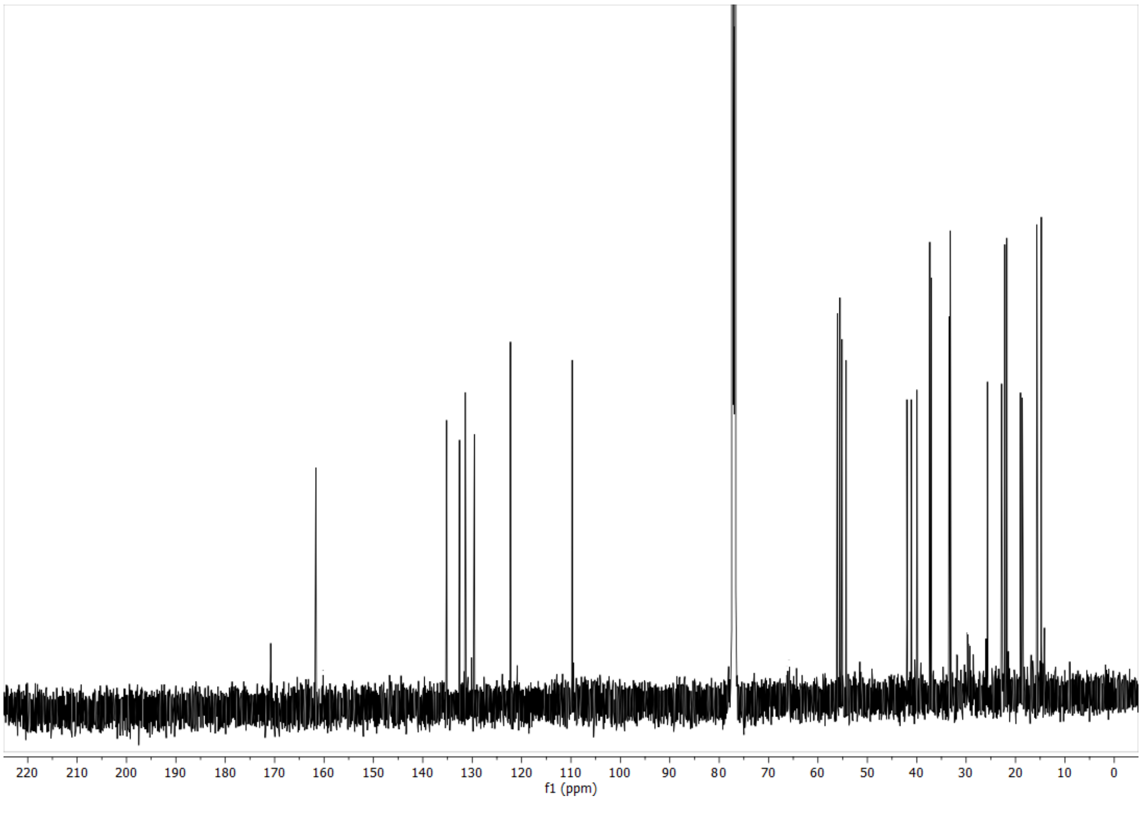

220
210
200
190
180
170
160
150
140
130
120
110
100
90
80
70
60
50
40
30
20
10
0
f1 (ppm)
